# Assessing bicycle safety risks using emerging mobile sensing data

**DOI:** 10.1016/j.tbs.2024.100906

**Published:** 2025-01

**Authors:** Yan Li, Yuyang Zhang, Ying Long, Kavi Bhalla, Majid Ezzati

**Affiliations:** a School of Architecture, Tsinghua University, Beijing, China; b School of Land Science and Technology, China University of Geosciences (Beijing), Beijing, China; c Department of Urban Planning and Landscape, North China University of Technology, Beijing, China; d School of Architecture and Hang Lung Center for Real Estate, Key Laboratory of Ecological Planning & Green Building, Ministry of Education, Tsinghua University, Beijing, China; e Department of Public Health Sciences, University of Chicago, Chicago, USA; f MRC Centre for Environment and Health, Imperial College London, London, UK

**Keywords:** Bike safety, E-bike, Infrastructure assessment, Machine learning, Street design

## Abstract

The surge in global electric bicycle ownership has exerted immense pressure on bicycle infrastructure. Theoretically, there’s a need to reassess the risk factors associated with multiple bike lane users. Based on this, there’s a practical need to re-evaluate the safety and quality of outdated infrastructure. This paper aims to reconsider risk factors related to bicycle infrastructure safety in the context of electric bicycles sharing lanes with traditional bicycles. Moreover, many countries lack precise spatial data concerning bicycle infrastructure. This study introduces a mobile sensing method based on bicycles, aiming to acquire daytime and nighttime bike lane datasets in a cost-effective, efficient, and large-scale manner. A computer vision-based bicycle risk factor assessment model was established, and the distribution of bicycle safety risk factors was visually analyzed. Research data was collected from a representative 59.5-kilometer bicycle lane area in Beijing. The results confirm the significant impact of the surge in electric bicycles, with electric bike users accounting for 72.1% of cyclists, 32.3% wearing helmets, and 8.4% riding against traffic. During the day, the highest-ranking risk factors include the type of bicycle lanes (half lacking dedicated lanes or being shared), roadside parking, and subpar road conditions. At night, insufficient street lighting are notable concerns. The research methodology is easily replicable and can be extended to new multi-user coexistence cycling environments or countries without bicycle spatial data, offering insights for bicycle safety policies and road design.

## 1 Introduction

Poor bicycle facilities have long been a safety concern and they can reduce the number of cyclists, thereby affecting population health. Cyclists are categorized as vulnerable road users because they lack the protection of a hard vehicle shell (Gatersleben and Appleton, 2007). Bicycle–motorized vehicle collisions account for the majority of recorded cyclist injuries and fatalities in police and hospital records. This is largely attributed to the absence of dedicated bicycle lane separation measures (Chong et al., 2010, Nicaj et al., 2009, Rosenkranz and Sheridan, 2003, Rowe et al., 1995, Sze et al., 2011).

Over the past decade, the use of electric bicycles (e-bikes) has surged globally, with (Table 1) China’s e-bike ownership reaching 300 million (https://www.gov.cn, 2020). This has placed additional pressure on infrastructure, necessitating the accommodation of various types of lane users. The commonly used push-style electric bicycles in China are both larger in size and heavier than traditional bicycles and are much faster. According to the new national standard (GB17761-2018), the center distance between the front and rear wheels of electric bicycles should not exceed 1.25 m, the width should not exceed 0.45 m, and the weight (including battery) should not exceed 55 kg. The speed should not exceed 25 km/h, but actual speeds can reach up to 70 km/h. In China, the law classifies electric bicycles as bicycles, requiring them to travel on bicycle lanes. This exacerbates safety risks for bicycle infrastructure and poses threats to traditional cyclists (Chataway et al., 2014, Daley and Rissel, 2011, Fishman et al., 2013, Gatersleben and Appleton, 2007, Heinen et al., 2010, Lawson et al., 2013, Rondinella et al., 2012, Schepers et al., 2014). In summary, while the size of bicycle lane users has grown, the design standards for bicycle infrastructure have not been updated accordingly. Whether outdated infrastructure can meet new usage demands remains an open question.

Consequently, in the context of multi-modal cycling interactions, a refined theoretical framework is imperative for assessing the safety and quality of infrastructure within the evolving bicycling ecosystem. Similar concepts for assessing cycling comfort and convenience have been proposed previously, such as bicycle level of service (Landis et al., 1997, Kazemzadeh and Ronchi, 2022), bikeability index (Winters et al., 2013), and Mobike Riding Index (Long and Zhao, 2020), and corresponding quantitative evaluation systems have been constructed. The abovementioned methods also attempt to assess bicycle infrastructure from a safety perspective, providing insights into constructing bicycle infrastructure safety risk factors. However, these conceptual frameworks have not yet incorporated the presence of electric bicycles.

From the perspective of cycling safety analysis methodologies and data availability, many countries, including China, lack detailed spatial data on cycling infrastructure, thereby hindering comprehensive evaluations of infrastructure safety. Accident data analysis is a prevalent approach, relying on publicly available accident reports and police records (Siman-Tov et al., 2018). However, acquiring such data can pose challenges. Road safety audits, on the other hand, involve on-site inspections of existing roads to assess potential hazards. While this method typically requires expert involvement, data acquisition is relatively straightforward (Nabors et al., 2012). With technological advancements, simulation studies (Nazemi et al., 2021) and GPS-based cycling data analyses (Saad et al., 2019) have become feasible, depending on specific tracking devices, virtual reality equipment, or advanced data analytics software. Conversely, questionnaire surveys (Calvey et al., 2015) and public engagement strategies (Sandt et al., 2015) offer researchers invaluable firsthand insights. In recent years, methods based on street-view imagery have gained traction. Commercial companies such as Google and Baidu provide application processing interface access to street view images (SVIs) covering multiple countries. Due to their consistency with the perspective of human eyes, SVIs have been used to evaluate cycling environments and environmental exposures (Eisenman et al., 2010). In addition, the development of computer vision techniques has catalyzed a recent surge in studies using SVIs, with machine learning-based image recognition and image segmentation models that reduce the cost of field research and improve the automation of analysis of built environments (Wang et al., 2019, Ito and Biljecki, 2021, Kang et al., 2020). Furthermore, inspired by commercial streetscape collection vehicles, mobile sensing approaches with humans and bicycles as agents are being gradually used by researchers for their flexibility and low cost and to expand the spatial coverage of data (Zhang, 2024a,b; Zhang et al., 2023), such as sidewalks and bicycle lanes not covered by commercial streetscapes with motor vehicles (Laport-López et al., 2020).

In this context, to address the pressing need for novel bicycle infrastructure safety assessments in the face of limited comprehensive spatial data, our objective is to develop a methodology for the acquisition of data pertaining to bicycle infrastructure safety. We have undertaken a case study in a data-scarce yet highly active bicycling environment situated in Beijing, China. This study is underpinned by the following specific objectives:

1. To formulate a comprehensive bicycle infrastructure safety risk factor system grounded in an extensive literature review and substantive workshop discussions;
2. To procure daytime and nighttime images of bicycle lanes through the utilization of a mobile sensing approach on the streets within a representative township;
3. To establish a risk factor recognition model based on computer vision technology. This model, on one hand, employs a deep learning-based object detection algorithm to identify infrastructure-related risk factors in daytime images and, on the other hand, assesses road surface luminance based on high dynamic range (HDR) imagery;
4. To evaluate the safety of the infrastructure on a per-street segment basis and furnish practical recommendations for street design enhancements within this evolving cycling environment.

## 2 Data and methods

### 2.1 Study area

**Fig. 1:**
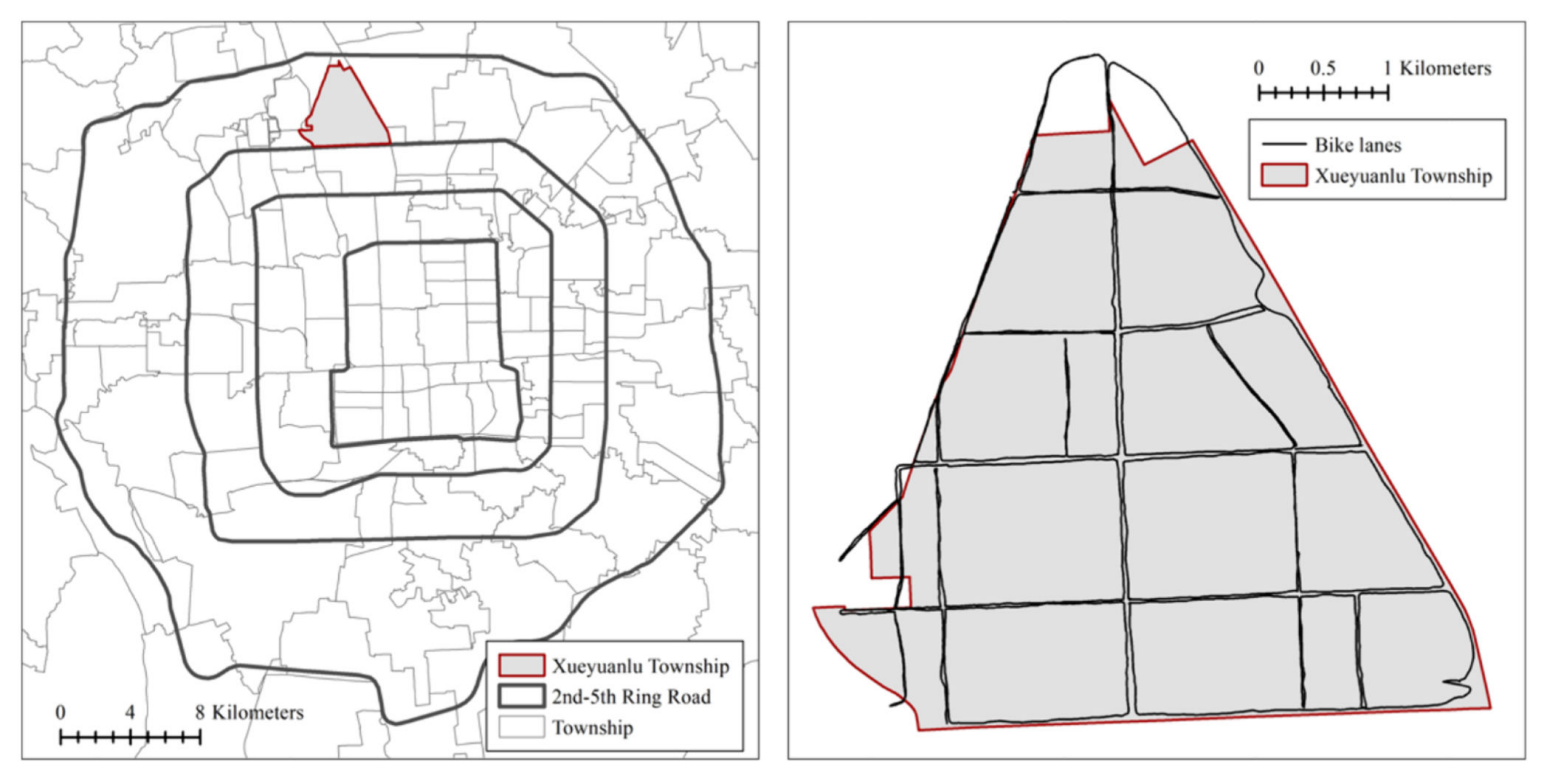
Xueyuanlu Township in Beijing as a study area.

In this investigation, Xueyuanlu Township in Beijing was deliberately selected as the focal region to exemplify the estimation model concerning infrastructure-related risk factors within bicycle lanes and overall bicycle safety in China (refer to Fig. 1). Xueyuanlu stands as a representative township within Beijing’s urban landscape, characterized by a diverse array of land uses, encompassing residential areas, commercial zones, and multiple university campuses. The study specifically concentrated on bicycle lanes situated along urban thoroughfares, excluding internal roads (e.g., paths within parks and gated communities). Urban roads were chosen due to their adherence to clear and standardized criteria, higher traffic volumes, and frequent interactions between bicycles and vehicles. Conversely, the design of internal roads often remains subject to the discretion of real estate developers and is characterized by greater flexibility.

The 2018 Amap road network data, a prominent mapping service in mainland China, served as the primary dataset for this investigation. Recognizing the outdated and incomplete nature of the data, we undertook a meticulous process of manual correction and supplementation, thereby underscoring the imperative need for fresh data collection efforts within this study. Notably, Xueyuanlu Township encompasses a land area of 13.7 km^2^, boasting an extensive network of 59.5 km of bicycle lanes.

### 2.2 Bicycle safety risk factors in Chinese cities

Preceding the commencement of measurements, an indicator system for assessing bicycle safety within Chinese cities was meticulously developed. The approach adopted in this study paralleled that put forth by Arellana for assessing walkability (Arellana et al., 2020), wherein factors and components were selected based on a comprehensive literature review. The questions for the literature review were as follows. Which risk factors are associated with bicycle infrastructure? Which factors are applicable to China? Which factors are detectable by mobile sensing?

To execute this, English literature from 2005 to 2021 was systematically retrieved from the Web of science and MEDLINE databases using keywords TS=(“exposure” or “built environment” or “environment” or “factor” or “risk factor” or “element” or “behavior” or “road” or “bike lane” or “motorway”) AND TS=(“moped” or “bicycle” or “e-scooter” or “scooter” or “rickshaw” or “bike” or “e-bike” or “electric bike” or “electric bicycle”) AND TS= (“review” or “systematic review” or “*meta*-analysis”) AND TS=(“safety” or “accident” or “collision”). This search yielded 72 literature reviews, out of which 65 were eliminated for their lack of relevance following title and abstract screening. Subsequently, six reviews remained after full-text scrutiny (detailed in **Appendix Table S1**). From these reviews, risk factors associated with infrastructure were extracted based on their propensity to contribute to collisions and falls involving motor vehicles or other bicycles.

Subsequently, infrastructure-related risk factors were established through a series of deliberations involving experts participating in the Pathways to Healthy and Equitable Cities project. This collaborative effort culminated in the creation of a comprehensive checklist encompassing a wide spectrum of risk factors within the global context.

Further insights were garnered from Chinese social media platform Weibo and Chinese court records to discern risk factors specific to Chinese cities. By analyzing historical data from Weibo spanning 2013 to 2016 and real-time data from 2021 using keywords TS= (“bike injuries” or “shared bike injuries” or “e-bike injuries”), issues related to bicycle infrastructure surfaced in user discussions. These included concerns such as uneven road surfaces, obstruction of views by trees or vehicles, ongoing road construction, and the absence of street lighting. Additionally, records from 2021 Beijing court judgments pertaining to bicycle traffic accidents and dangerous driving offenses were scrutinized. The primary categories of accidents included collisions involving bikes/shared bikes/e-bikes with cars (most prevalent), bikes/shared bikes/e-bikes with pedestrians, and bikes/shared bikes colliding with e-bikes. Notably, bicycle injuries and fatalities were predominantly concentrated at road intersections (with right-turning cars at intersections without traffic light controls posing a particular concern), mixed-use roads (where motorized and non-motorized lanes were not segregated), and narrow roads. The result of this meticulous process culminated in a refined list comprising 17 factors that aptly represented indicators for bicycle infrastructure safety within the Chinese context.

Lastly, for each of these factors, an assessment was made regarding their visual representation and identifiability within images, accompanied by sample images for reference (as depicted in Table 2).

The type of bicycle lane significantly influences the potential interactions between bicycles and motorized vehicles. Four distinct categories are identified in China: separated lanes with fixed barriers like green belts or sidewalks, protected lanes with removable guardrails, painted lanes with delineated lines, and the absence of dedicated lanes where cyclists share road space with motorized vehicles. From a safety perspective, separated bicycle lanes with physical barriers effectively eliminate the possibility of contact between bicycles and motor vehicles (Poudel and Singleton, 2021). Conversely, the absence of dedicated bicycle lanes places cyclists in direct exposure to potential motor vehicle interactions, significantly increasing the likelihood of contact and collisions.

Regarding road segment configurations, narrow bicycle lanes lacking segregation measures tend to lead cyclists to inadvertently cross into motor vehicle lanes, thereby elevating the risk of collisions (Thomas and DeRobertis, 2013, Vanparijs et al., 2015, DiGioia et al., 2017). Inadequate street lighting can result in cyclists colliding with motor vehicles, other bicycles, or infrastructure elements due to compromised visibility of road conditions (Reynolds et al., 2009, Vanparijs et al., 2015, Prati et al., 2018, DiGioia et al., 2017). The presence of bus stops within bicycle lanes poses a collision risk, as cyclists may come into contact with large buses that occupy the bicycle lane during stops. Similarly, trucks, often of comparable or larger size than buses, may disregard cyclists due to their high inertia and extensive blind spots, thus presenting a substantial hazard to cyclists (Prati et al., 2018). Incidents involving on-street parking and lane occupation can result in constrained bike lane space, potentially leading to accidents as bicycle and e-bike users may collide with car doors upon opening (DiGioia et al., 2017). The presence of entrances within bicycle lanes can also precipitate collisions due to motor vehicle blind spots during turning maneuvers (Prati et al., 2018). The presence of entrances within bicycle lanes can also precipitate collisions due to motor vehicle blind spots during turning maneuvers (Reynolds et al., 2009, Prati et al., 2018). Additionally, maintaining a consistent elevation between the bicycle lane and the sidewalk can widen the shared space for pedestrians, bicycles, and e-bikes, potentially leading to increased collision rates (Poudel and Singleton, 2021).

With respect to road conditions, road friction plays a crucial role in determining a cyclist’s ability to maintain control, brake effectively, and navigate safely, especially in challenging conditions like wet or icy surfaces (Schepers and Wolt, 2012). Steep inclines or declines can significantly affect cycling safety, particularly when combined with other risk factors (Dozza and Werneke, 2014).

As for the road environment, the volume and speed of nearby vehicular traffic can substantially impact cyclist safety and comfort (Jacobsen et al., 2015). Ambient noise levels. Additionally, high ambient noise levels can mask crucial auditory cues that cyclists rely on for safety (Zhang et al., 2023).

The configuration of intersections also plays a vital role in cycling safety. Dedicated left-turn bike lanes at intersections significantly reduce the risk of collisions between cyclists and vehicles during left turns, a common point of conflict in urban cycling (Monsere et al., 2014). Bicycle-specific traffic signals enhance safety by providing clear, targeted guidance to cyclists, thereby reducing confusion and potential conflicts with vehicular traffic, especially during turn phases (Thompson et al., 2013). Bicycle Waiting Areas (also known as Bike Boxes) increase cyclist visibility and provide a designated safe space at intersections, reducing the risk of right-hook collisions and allowing cyclists to safely position themselves for left turns (Dill et al., 2012).

### 2.3 Mobile sensing

The daytime data collection took place on March 6, 2022, with clear weather, temperature −1 to 11 degrees Celsius, and the collection activity took place from 14:52 to 17:46, lasting 2 h and 54 min. The nighttime data collection took place on May 22, 2022, with clear weather, temperature from 1 to 9 degrees Celsius, and the collection activity lasted from 20:12 to 23:15, lasting 3 h and 3 min. Both sessions were conducted during spring (March to May) in Beijing on Sundays. Sunny days were chosen to ensure the clarity of the captured images. We deliberately avoided peak travel hours to better capture risks associated with street infrastructure and ensure the safety of data collectors. In this research, the acquisition of research data involved the compilation of daytime and nighttime mobile sensing datasets, encompassing four distinct phases: route planning, camera installation, data collection, and data processing. In this research, the acquisition of research data involved the compilation of daytime and nighttime mobile sensing datasets, encompassing four distinct phases: route planning, camera installation, data collection, and data processing. However, variations in the daytime and nighttime environments necessitated the utilization of different camera equipment and data processing techniques. Firstly, route planning was undertaken to ensure comprehensive coverage of all bicycle lanes with minimal time expenditure, while maintaining consistency between daytime and nighttime travel routes. Chinese postman algorithms were employed to derive the most efficient routes. Secondly, the establishment of a camera system was imperative to encompass the evaluation areas effectively. A GoPro 9 Black camera was deployed for daytime recording, capturing infrastructure facing the bicycle lane, while a Sony DSLR ILCM-7AM3A camera was utilized during nighttime operations to ensure optimal clarity for the acquisition of road surface luminance data. Thirdly, during the data collection phase, the pre-defined navigation routes were meticulously followed. The cameras were activated, and a dedicated e-bike maintained a constant speed of 25 km/h to capture corresponding street scenes. Each trip culminated in the retrieval of GPS trajectories and camera data. Finally, the data processing endeavor was centered on transforming the collected information into a uniformly distributed, georeferenced dataset of bicycle lane images.

#### 2.3.1 Daytime bicycle lane image collection

The objective of daytime data collection was to comprehensively document the infrastructure encompassing all bicycle lanes and side-walks within the designated study area. Initially, given the unidirectional nature of bicycle lanes in China, it was imperative to ensure that each road segment covered both left and right directions, thereby achieving complete spatial coverage of all bicycle lanes. To optimize efficiency and minimize time expenditures, the route planning phase leveraged ArcPy code integrated with ArcMap’s Network Analyst tool, resulting in the generation of a 59.5 km navigation path, which was subsequently stored in.kml format.

**Fig. 2:**
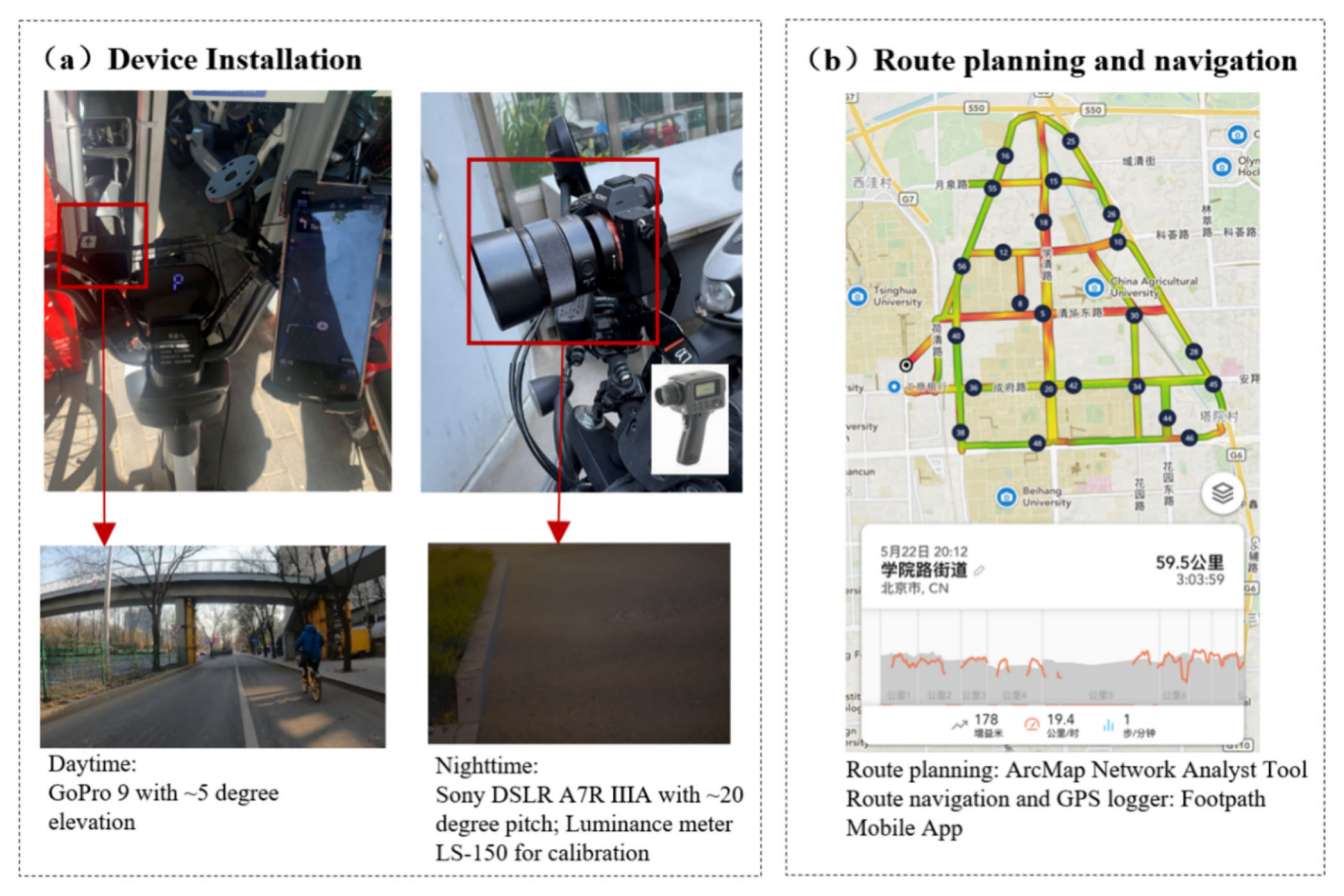
Mobile sensing platform for street-level data collection.

During the data collection process, the “Footpath” mobile application was employed to import the pre-planned route and initiate navigation while concurrently recording the GPS track of the data acquisition route. This endeavor was executed utilizing a dedicated e-bike, specially equipped with a GoPro 9 Black camera mounted on the handle, oriented to capture the bicycle lane ahead at a 5° elevation angle (refer to Fig. 2). To facilitate uninterrupted data collection, each camera was supplied with an additional battery, supplementing its primary power source, to ensure the planned 3-hour data collection duration. Each battery had the capacity to sustain 1.5 h of recording with the screen deactivated. The camera settings were configured to a standard resolution of 1920 × 1080 pixels. The dedicated e-bike maintained a consistent speed of 25 km/h throughout the data collection process.

In the subsequent data processing phase, given that GPS coordinates were logged at one-second intervals, accompanied by corresponding timestamps and geographic coordinates, individual image frames were extracted from the video footage at one-second intervals, with concurrent timestamp recording to ensure uniform spatial resolution. Building upon this foundation, geographic coordinates were aligned with their corresponding images based on matching timestamps. To address redundancy stemming from traffic light stops, an image similarity detection mechanism was applied to eliminate images exhibiting more than a 70 % similarity threshold. All aspects of data processing were executed through Python, utilizing the image processing capabilities of OpenCV, in conjunction with data processing libraries, Pandas and NumPy.

#### 2.3.2 Nighttime bicycle lane image collection

The primary objective of nighttime mobile sensing operations was to capture the luminance characteristics of the road surface, particularly in correspondence with street lighting conditions. To facilitate spatial analysis on a consistent scale, the nighttime data acquisition route and navigation procedures were identical to those employed during daytime operations.

In terms of equipment setup, the nighttime conditions, characterized by reduced visibility, necessitated the deployment of a high-definition stabilized camera capable of accommodating longer exposure times to ensure image clarity. Accordingly, a Sony DSLR A7R IIIA camera was selected, operating in time-lapse mode rather than video mode, as nighttime video quality tends to be suboptimal, and High Dynamic Range (HDR) images cannot be effectively generated. Photos were captured at one-second intervals and stored in RAW format. To capture the luminance values of the road surface accurately, the camera was securely mounted on the e-bike’s handlebars, oriented to face the road surface of the bicycle lane directly ahead, with a pitch angle of 20°.

For the subsequent conversion of photographic data into luminance values, a luminance meter (LS-150) was employed to measure luminance at five distinct sampling points. These sampling points were chosen at well-defined, sharp-edged corners, each characterized by varying levels of illumination to facilitate calibration. Corresponding photographs of these sampling points were also collected for reference. In the data processing phase, the coordination and photographs were correlated and subjected to deduplication, consistent with the timestamp-based methodology employed during daytime data processing.

### 2.4 Measurement of bicycle infrastructure risk factors

#### 2.4.1 Identification of daytime risk factors

The identification of daytime risk factors primarily relied on image analysis facilitated by deep learning-based object detection algorithms. Initially, for the four distinct types of bicycle lanes—separated, protected, painted, and no bicycle lanes—characterization was based on the presence of fixed barriers, removable barriers, or white lines. Subsequently, feature objects corresponding to these delineations were automatically detected through the utilization of an object detection algorithm, subsequently allowing for their classification. The absence of any feature object in a given segment indicated the absence of a designated bicycle lane, consequently leading to its categorization as such. It was assumed that each street segment pertained to a specific street type, and if at least one feature object was detected within that segment, the street was classified accordingly. Likewise, the remaining six risk factors—namely, the presence of bus stops, on-street parking, objects obstructing bicycle lanes, entrances and exits, suboptimal road-surface conditions, and uniform elevation between the sidewalk and bicycle lane—were identified through the detection of corresponding feature objects within the images. The object detection model proved effective in identifying these phenomena, encompassing bus stops, motor vehicle parking, small vehicles and obstructions occupying roadway space, road surface imperfections such as potholes and cracks, consistent pavement edge elevations, and zebra lines indicative of entrances and exits. For preparing the training dataset for the automatic deep learning model, each object was meticulously labeled with a rectangular bounding box, utilizing the Python-encoded LabelImg toolkit. Given the varying size, shape, and quantity of objects in each image, the dataset poses challenges in detection and underscores the importance of a comprehensive training dataset.

In light of the identified and labeled objects, the automatic recognition model was developed employing a Convolutional Neural Network (CNN), chosen due to its exceptional performance surpassing human recognition capabilities in image object detection tasks. Particularly, the YOLOv7 architecture has demonstrated remarkable success in this regard (Wang et al., 2022). The implementation of the detection model was executed using PyTorch, and it was trained from scratch using the MS COCO datasets to detect the aforementioned 12 categories of objects. To ensure robustness and mitigate issues related to overfitting and underfitting, cross-validation was employed for evaluating the performance of the machine learning model. In the cross-validation process, the original dataset was partitioned into 10 distinct subsets, with nine of these subsets utilized for training the model, while the remaining subset was reserved for testing. This procedure was iterated 10 times, with a different subset designated as the test set in each iteration. Subsequently, performance metrics obtained from each individual test were aggregated to derive the final performance metric for the model. For both training and testing phases, the input images retained their original dimensions of 1920 × 1080 pixels without undergoing any preprocessing steps. The optimization process was conducted through the stochastic gradient descent solver, spanning 100 iterations, with an initial base learning rate of 0.001. Subsequently, an additional 100 iterations were executed, accompanied by a reduction in the base learning rate to 0.0001.

Subsequent to the classification of bicycle lanes, the determination of their respective widths was undertaken. Initially, the bicycle lanes were delineated within the images through the utilization of the SegNet model (Badrinarayanan et al., 2017), resulting in the segmentation of multiple pavement segments. However, due to the perspective originating from a bicycle-centric vantage point, the central pavement was singled out for further analysis. In the subsequent step, camera calibration techniques were applied to facilitate perspective transformation, thereby converting image pixel coordinates into corresponding geographic locations. This process rectified the distortion observed in roads, particularly the narrowing effect as the viewpoint receded, ultimately restoring them to parallel alignment. Ultimately, the Python Shapely package’s “line1.distance (line2)” function was employed to calculate the width of the bicycle lanes by measuring the distance between the edges of the roadway.

For road condition identification, we adopted a deep learning-based approach to estimate road friction coefficients (Roychowdhury et al., 2018). Specifically, we developed a modified ResNet-50 model that analyzes 224x224 pixel road surface images. The model classifies road surfaces as dry, wet, snowy, icy, or oily, and estimates friction coefficient ranges accordingly (e.g., dry: 0.7–0.9, wet: 0.4–0.7, snowy: 0.2–0.4, icy: 0.1–0.3, oily: 0.1–0.2) (Hall et al., 2009). This method enables large-scale, continuous road condition monitoring utilizing existing traffic camera data. However, since all sampled roads were dry, there were insufficient samples for classification, and due to the lack of variability, this indicator was not included in the subsequent results.

To estimate road gradients, we leveraged high-precision GPS data points along the road, encompassing longitude, latitude, and altitude information. After preprocessing to remove outliers, we associated GPS coordinates with road sampling points using spatial correlation. Subsequently, we projected the coordinates to the WGS1984_50N coordinate system, calculated the horizontal distance between adjacent points, and computed the altitude difference. Finally, the local gradient between adjacent sampling points was calculated as (altitude difference / horizontal distance) * 100 %.

Regarding the traffic environment, we employed a random forest model (R2 = 0.72, RMSE=3.28 dB) to predict traffic noise, based on Zhang et al.’s research (Zhang et al., 2023). Our approach involved collecting street view images and built environment data. Utilizing a YOLOv7 model and GIS analysis tools, we measured 49 predictor variables, including microscopic traffic composition, street form, and land use, to obtain traffic noise values for each sampling point.

The quantification of bicycle traffic flow primarily relies on object detection and tracking algorithms in computer vision technology. Initially, the deep learning model YOLOv7 was employed to process video frames, identifying and locating bicycles and electric bicycles in each frame. Subsequently, an object tracking algorithm, DeepSORT (Wojke et al., 2017), was implemented to track the movement of each identified vehicle across consecutive frames, assigning a unique ID to each traffic entity. The number of unique IDs is tallied for each sampling point image; to calculate the flow for each road segment, we aggregate the number of IDs from all samples on that road.

With respect to intersection analysis, our current study area lacks specialized configurations for cyclists at intersections, resulting in insufficient samples for implementing automatic recognition based on deep learning methods.

#### 2.4.2 Identification of nighttime risk factors

When assessing nighttime illumination levels, High Dynamic Range (HDR) photography serves as a valuable tool for luminance measurement. In accordance with the guidance provided by Kumaragurubaran (Kumaragurubaran and Inanici, 2013), the initial step involved the conversion of raw photographs into the HDR format, a task accomplished utilizing Python’s OpenCV toolkit. During this conversion process, particular attention was given to maintaining consistent color space transitions, necessitating the adjustment of white balance and contrast settings. An aperture size of f/4.0 was consistently employed, complemented by a shutter speed of 1/100 sec. The resulting HDR images were stored in.hdr format, allowing for pixel values to span the luminance range perceivable by the human visual system, spanning 14 logarithmic units, from 10.8 to 106 cd/m^2^. Subsequently, these converted HDR images underwent calibration, a process that involved the inclusion of five sampling points and an analysis procedure to derive per-pixel luminance data. The HDRSCOPE software was instrumental in facilitating this calibration process. Subsequent to the generation of the luminance map, the NumPy toolkit for Python was leveraged to calculate the average luminance values for the images, contributing to a comprehensive evaluation of nighttime lighting conditions.

#### 2.4.3 Measurement of safety at street level

The outcomes pertaining to risk factors at the individual point level were aggregated at the street level following identification through the computer vision methodology, as outlined in Table 3. In this tabulation, higher values are indicative of elevated risk levels. The hierarchical arrangement of road risk levels progressed in ascending order from separated bicycle lanes to scenarios without dedicated bicycle lanes, resulting in categorized values ranging between 0 and 3. The assessment of insufficient street lighting was conducted by computing the average illuminance across all points within a given street segment. If the calculated average illuminance surpassed 0.5, the corresponding risk level was assigned a value of 0; conversely, if the average fell below 0.5, a risk level of 1 was assigned, consistent with the criteria established by Kostic and Djokic (2012). Conforming to the Planning Standards for Urban Pedestrian and Bicycle Transportation Systems (No. GB/T51439-2021), risk values were determined for bicycle lane width. Specifically, if the average width of bicycle lanes across all street sections was less than 1.5 m, a risk value of 1 was designated; conversely, if the average exceeded 1.5 m, a risk value of 0 was assigned. The assessment of the bus stop factor entailed the calculation of bus stop density within the street segment, measured as the number of bus stops per kilometer. Additionally, six other indicators were evaluated using a density-based calculation approach. These included on-street parking, objects obstructing bike lanes, the presence of entrances, adverse road conditions, uniform elevation levels between the sidewalk and bicycle lane, and the presence of large vehicles.

## 3 Results

### 3.1 Mobile sensing datasets

The daytime dataset comprises a GPS-recorded route in.gpx format, spanning 59.5 km in length, and lasting for 2 h and 54 min. Additionally, it includes 15 video files in.avi format, each capturing segments of the route (GoPro cameras automatically generate videos every 12 min). Through data processing, a total of 8,251 images were extracted, each geo-referenced at approximately 7-meter intervals, which corresponds to a time interval of 1 s at a vehicle speed of 25 km/h.

Conversely, the nighttime dataset encompasses 59.5 km of orbital data in.gpx format, extending over a duration of 3 h and 3 min. It also incorporates nighttime photographs taken at 1-second intervals, saved in.raw format. Post-processing yielded a collection of 5,770 nighttime photos, each accompanied by geographic coordinates.

In comparison to commercial Street View Imagery (SVI) datasets, such as those offered by Google and Baidu, and other publicly available SVI datasets like the Cityscapes dataset, our dataset exhibits several distinctive attributes:

- Bicycle-centered Perspective. Diverging from the motor vehicle-oriented viewpoints prevalent in existing datasets, our dataset captures comprehensive images of the entire bicycle lane. To the best of our knowledge, it stands as the first street view dataset obtained from the vantage point of a bicycle lane.
- Day and Night Coverage: This dataset not only encompasses daytime conditions of bicycle lanes but also gathers nighttime street videos and lighting data. This inclusion addresses the challenge of nocturnal data collection, where lighting is often inadequate.
- Extensive Sample Size: Encompassing 59.5 km of bicycle lanes, the dataset incorporates geo-referenced video data, 8,251 daytime images, and 5,770 nighttime images.
- High-Resolution Imagery: Both daytime and nighttime images were acquired at a high resolution of 1920 × 1080 pixels, facilitating precise object identification.
- Comprehensive Bicycle-Related Labels: For object detection purposes, the labeled dataset comprises 1,789 images encompassing a total of 40,164 labeled objects. These objects span a wide range, including fixed and removable barriers, objects obstructing bicycle lanes, pedestrian crossing signs, bicycle landmarks, bicycle road markings, road cracks, bus stops, bicycle lane underlays, uniform elevation between the bicycle lane and sidewalk, zebra crossings, on-street parking, pavement guardrails, and trucks.

Leveraging these distinctive attributes, we conducted an extensive series of analyses pertaining to bicycle safety risk factors.

### 3.2 Accuracy of measurement models

The measurement of risk factors was completed using three computer vision models, and therefore, the models were validated separately. The first model is the object detection model based on YOLOv7 with mean Average Precision (mAP) metric for validation. The mAP score for all categories was 0.82, of which on-street parking obtained the highest score of 0.92 and mobile barriers obtained the lowest score of 0.73. This reveals that using the YOLOv7 model to detect risk factors is highly effective. The second model is lane detection and camera correction. The lane detection model was trained using the existing segmentation model SegNet, which achieved an accuracy of 96.4 % for the road category (Badrinarayanan et al., 2017). To validate the road width after camera calibration, we randomly selected five road segments to compare the measured values with the ground truths and calculated the error using the Root Mean Square Error (RMSE) metric, resulting in a value of 0.51 m, indicating satisfactory results. The last model is luminance calculation based on HDR images. According to the accuracy reported in the adopted method (Kumaragurubaran and Inanici, 2013), the luminance measurement error was less than 0.02 cd/m^2^.

### 3.3 Bicycle safety mapping

The statistical analysis of ten risk factors for 124 bicycle lanes in the study area is presented in Table 4. The average illuminance of all lanes is 0.463 cd/m^2^, which falls below the road lighting standard requirement of 0.5 cd/m^2^. The minimum width value for all roads is 1.469 m, closely aligning with the design standard regulation of 1.5 m, and can be considered as meeting the requirement. The maximum road width value is 10.705 m, with an average width of 4.419 m. On-street parking, adverse pavement conditions, and road occupation exhibit the highest incidence, with an average of 20.681, 14.478, and 4.904 occurrences for all roads, and 87.125, 50.615, and 20.917 occurrences per square kilometer for the most problematic roads. Conversely, entrances and large vehicles exhibit the lowest occurrences, with an average of 1.269 and 1.595 occurrences per square kilometer for all roads.

**Fig. 3:**
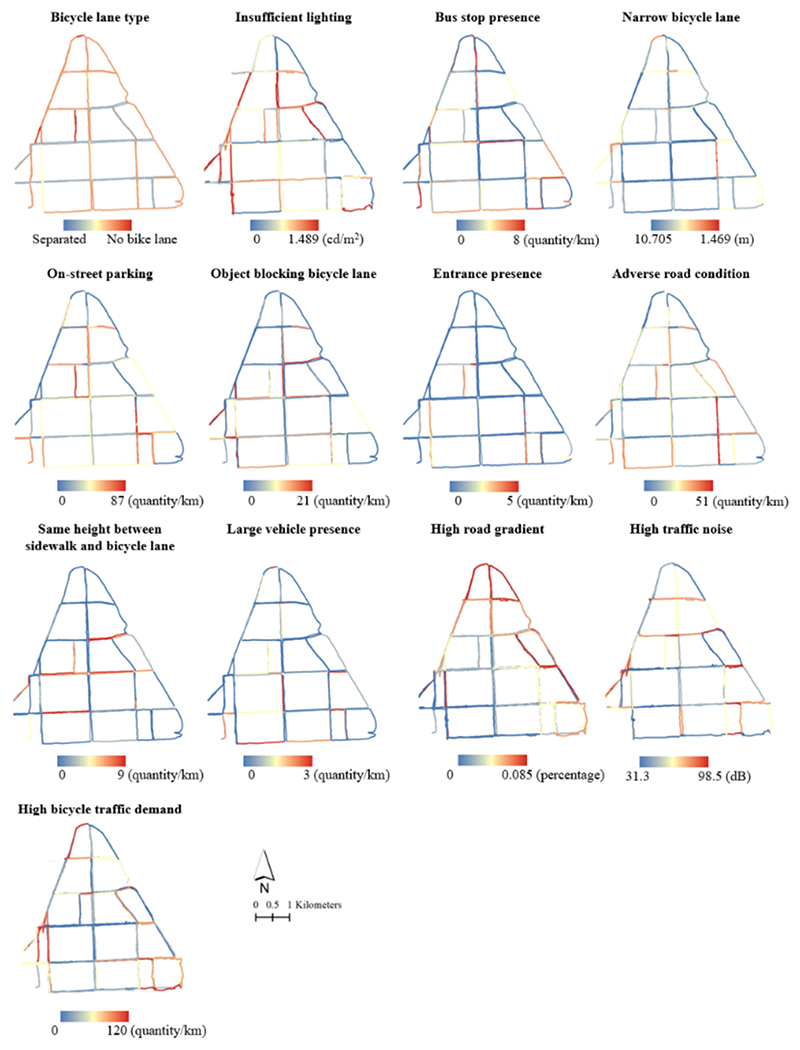
Spatial distribution of infrastructure-related risk factors.

The bicycle safety map, illustrated in Fig. 3, presents a visualization of each factor in quintiles, with road colors transitioning from red to blue, indicating increasing levels of safety. The study area encompassed all types of bicycle lanes, with “no bicycle lane” being the shortest, spanning 2.37 km, and “painted bicycle lane” being the longest, covering 26.97 km. Additionally, “separated bicycle lanes” and “protected bicycle lanes” extended over 16.60 km and 4.68 km, respectively. The predominant bicycle lane type for east–west roads was “separated bicycle lanes”, while the roads located at the boundary of the study area were primarily characterized by “painted bicycle lanes”, as they constituted higher-class arterial roads. Urban roads surrounding the neighborhoods, devoid of dedicated bicycle lanes, accounted for a minor proportion of the urban road network, totaling 14 segments in total.

Among all the identified risk factors, the most prevalent and severe ones were on-street parking, adverse road conditions, and insufficient street lighting, affecting approximately 65 %, 64 %, and 88 % of the bicycle lanes, respectively. Conversely, the risk factor with the most favorable conditions was the presence of entrances, occurring on only 20 % of the roads, followed by consistent elevation between the side-walk and bicycle lanes, which was observed in 23 % of the lanes. On-street parking and adverse pavement conditions are pervasive issues. For instance, in residential areas, parking space may be limited, on-street parking might be unrestricted, or dedicated parking spaces may be insufficient, leading to frequent challenges for bicycle safety. This is especially evident when motor vehicles enter and exit parking spaces or when doors are opened, which can pose significant risks to cyclists. Instances of obstructions within bicycle lanes are primarily concentrated in two commercial areas characterized by high-density shops and sub-way entrances. In these areas, shared bicycles and delivery vehicles are often parked haphazardly. These zones experience heavy foot traffic, resulting in limited road space for parking, leading many individuals to park their bicycles within the bicycle lanes. Additionally, the prevalence of food delivery riders exacerbates the issue, further compressing the space available for safe bicycle travel. The map also highlights areas with high bicycle traffic demand, where red represents segments with up to 120 bicycles per kilometer, emphasizing critical zones for safety considerations. Additionally, it shows the distribution of traffic noise, with red indicating levels as high as 98.5 dB, which could impact cyclist safety and comfort. The road gradients in the study area were low, less than 0.085 %, and would not affect cyclist exertion and stability. Furthermore, our study revealed that only 11 street segments complied with nighttime road lighting standards, which is detrimental to bicycle safety during nighttime hours. Notably, motor vehicle lanes generally enjoyed better nighttime lighting coverage, while bicycle lanes often remained unilluminated due to the shading effect of street tree canopies.

### 3.4 The clustering results of risk factors

To demonstrate the situation of each risk factor category, we utilized hierarchical clustering to categorize the model identification results. This approach allows for control of different categories, such as various types of large vehicles, different colors of e-bike riders, and items obstructing the bike lane. Additionally, it provides a better understanding of the risk level, including potholes on the road with varying degrees of settlement. Only the results of identifying daytime risk factors are clustered here and displayed in Table 5, as only pictures of the pavement taken at night with brightness are available.

### 3.5 The threat of e-bikes

The study also recognizes the distinct category of e-bike riders, whose prevalence has surged, thereby exerting additional stress on bicycle infrastructure (Gu et al., 2021). In China, the ownership of e-bikes has surged to 300 million (https://www.gov.cn, 2020). We employed the same object detection model as described in Section 2.4 for this purpose. The model was trained based on labeled categories, including typical e-bike users, couriers, compliance with helmet usage, and adherence to traffic regulations, all discerned within the sample images. The findings revealed a greater prevalence of e-bike users compared to conventional cyclists in the bicycle lane, constituting 72.1 % of all users within the daytime dataset. Among the e-bike users, 62.3 % were identified as couriers involved in food and small item deliveries. They were often distinguished by their distinctive uniforms, frequently characterized by the colors associated with their courier companies (such as yellow, blue, and green), and equipped with food delivery boxes affixed to the rear seat. Notably, courier companies typically implement performance-based incentive systems, which encourage couriers to maximize their speed to secure more orders. Consequently, this incentive structure leads to distinct behaviors among couriers in contrast to regular users, making them more prone to traffic rule violations (Wang et al., 2021).

In China, the most common type of e-bike is the hand-throttle variety, which is limited to a speed of 25 km/h in accordance with the new national standard (GB 17761–2018). We conducted a survey on the public’s perception of e-bike speeding behavior using an anonymous questionnaire distributed through the social media platform WeChat. The survey comprised five main sections: participant age and gender, type of bicycle used for daily riding (traditional or e-bike), riding speed range, and attitudes towards e-bike safety and concerns. A total of 30 e-bike riders and 30 conventional bike riders were recruited to share and evaluate their experiences. No additional surveys were conducted once the required number of participants was reached. The study participants were primarily college students, as the research was limited to social networks. It is worth noting that all e-bike users reported exceeding the stipulated speed limit on a daily basis, with an average speed of 42 km/h. Additionally, 28 out of 30 traditional bike riders (93 %) expressed concern about the potential risks posed by e-bikes during their daily rides. Of particular concern is the inadequate accommodation for parking electric delivery bikes, which are frequently parked haphazardly, encroaching upon bicycle lanes and even pedestrian walkways, thereby causing inconvenience to pedestrians. The video footage captured during the study revealed instances where electric delivery bikes, faced with insufficient parking facilities at crowded restaurant locations, resorted to parking in motorways while awaiting meal pickups.

Inappropriate behaviors of e-bike users, such as not wearing helmets and riding in the wrong direction, were also observed during the study. These behaviors not only impact the safety and health of e-bike users themselves but also pose risks to other road users. Head injuries constitute the most prevalent type of injury among e-bike users (Li et al., 2022), and since June 1, 2020, Beijing has mandated helmet use for e-bike riders (excluding conventional bicycle users). However, as of May 22, 2022, when the study data was collected, the compliance rate for daily travelers wearing helmets remained relatively low, at 32.3 %. Riding in the wrong direction is another common behavior, accounting for 8.4 % based on our observations. Further investigation into additional cycling behaviors may be warranted in future studies.

**Fig. 4:**
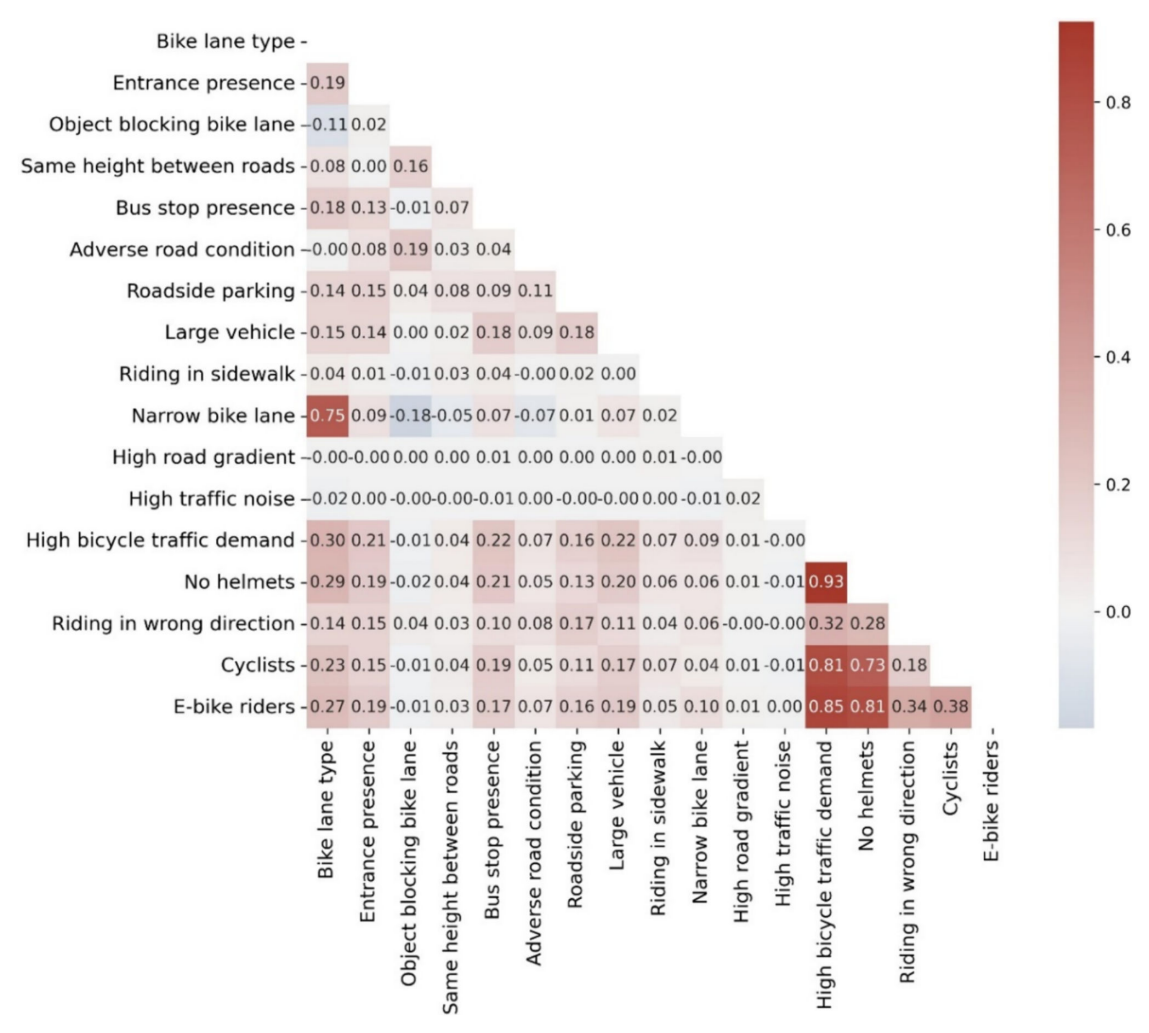
The correlation between risk factors and cycling behavior.

The correlation coefficient matrix was utilized to analyze the correlation between cycling behavior and risk factors using Pearson’s correlation coefficient (see Fig. 4). In areas with multiple entrances, bus stops, and large vehicles, there were more riders, with correlation co-efficients of 0.15 or higher. The correlation between not wearing helmets and high bicycle traffic demand is remarkably strong (0.93), indicating that in areas with elevated bicycle traffic demand, there is a significantly higher proportion of riders who do not wear helmets. Traditional cyclists, in particular, exhibit a higher incidence of helmet non-compliance, as evidenced by a strong positive correlation (0.85) between cyclists and the lack of helmet usage. Furthermore, e-bike riders demonstrate a notable correlation with both helmet non-compliance and riding in the wrong direction (0.38 and 0.32, respectively), suggesting that these unsafe behaviors are more prevalent among e-bike users. Additionally, there is a positive correlation (0.28) between riding in the wrong direction and helmet non-compliance, indicating that helmet non-compliance is also common among riders who engage in wrong-way riding.

## 4 Discussions

### 4.1 Comparison with other data sources

**Fig. 5:**
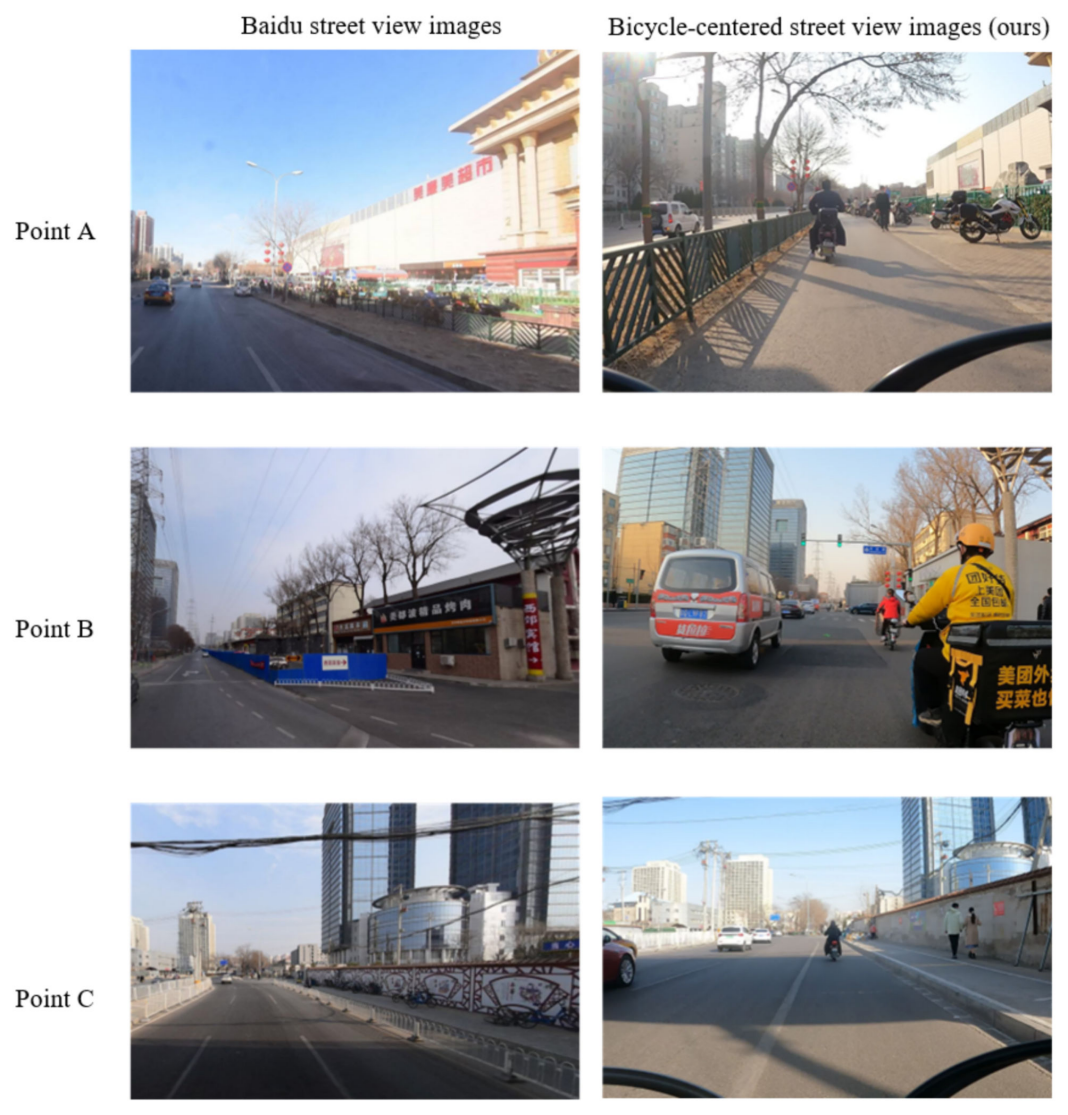
Comparison between Baidu SVIs and bicycle-centered SVIs (ours).

We collected street view images to describe bicycle lanes and measure infrastructure-related risk factors. Previously, similar empirical studies have been conducted using open-source commercial SVI data, such as Google SVI (Ito and Biljecki, 2021). Fig. 5 illustrates the primary rationales for utilizing self-collected street view data as opposed to commercial SVI data. Firstly, commercial SVI data is typically captured from motorways and thus fails to provide insights into the conditions of bicycle lanes, particularly those featuring a dedicated barrier for separation. Secondly, commercial SVI data tends to be outdated, lagging in its ability to reflect the most recent developments in the urban built environment, which is often characterized by rapid transformations. For instance, new road cracks and potholes frequently emerge, rendering the data obsolete. Thirdly, commercial SVI data exhibits lower spatial resolution, resulting in reduced clarity when it comes to depicting bicycle lane infrastructure. Furthermore, the resolution of the data is insufficient to enable clear differentiation of these elements.

Point A: Commercial street views are unable to provide insights into the conditions of separated bicycle lanes. Point B: Bicycle infrastructure within the city undergoes rapid changes, while commercial street views often lag behind in updating to reflect the most current conditions. Point C: Commercial street views lack the ability to depict specific conditions of bicycle lanes, even in scenarios without any obstructions. They are inadequate for assessing risk factors related to road cracks and lane width.

### 4.2 Image source: mobile sensing images

We compared the results from the study area with machine learning results based on commercial SVI. SVI images from Baidu, China’s largest street view service provider, were downloaded based on the same sampling points but with some limitations such as motorized vehicle viewpoints, inconsistent age (2013–2022), and poor image clarity (see Table 6). By comparing the results (see Table 4), we found that the machine learning recognition method based on commercial SVIs fails to identify nighttime risk factors and underestimates all daytime risk factors, except for on-street parking (which is more highly recognized by Baidu) and presence of entrances (both of which are roughly the same). Our method has higher recognition values for adverse pavement conditions, same height between bike lane and sidewalk, large vehicle presence compared to commercial SVIs. It can be seen that the risk factors were greatly underestimated if the existing commercial SVI based approach was used.

Simultaneously, the data collection process necessitates physical surveys of both sides of each street segment, a task demanding extensive human labor. This study explores the use of mobile sensing data for measuring bicycle infrastructure and establishes the viability of this approach. In the future, the development of a crowdsourcing image platform could enable the collection of street-level images encompassing all street segments within the Beijing urban area.

**Fig. 6:**
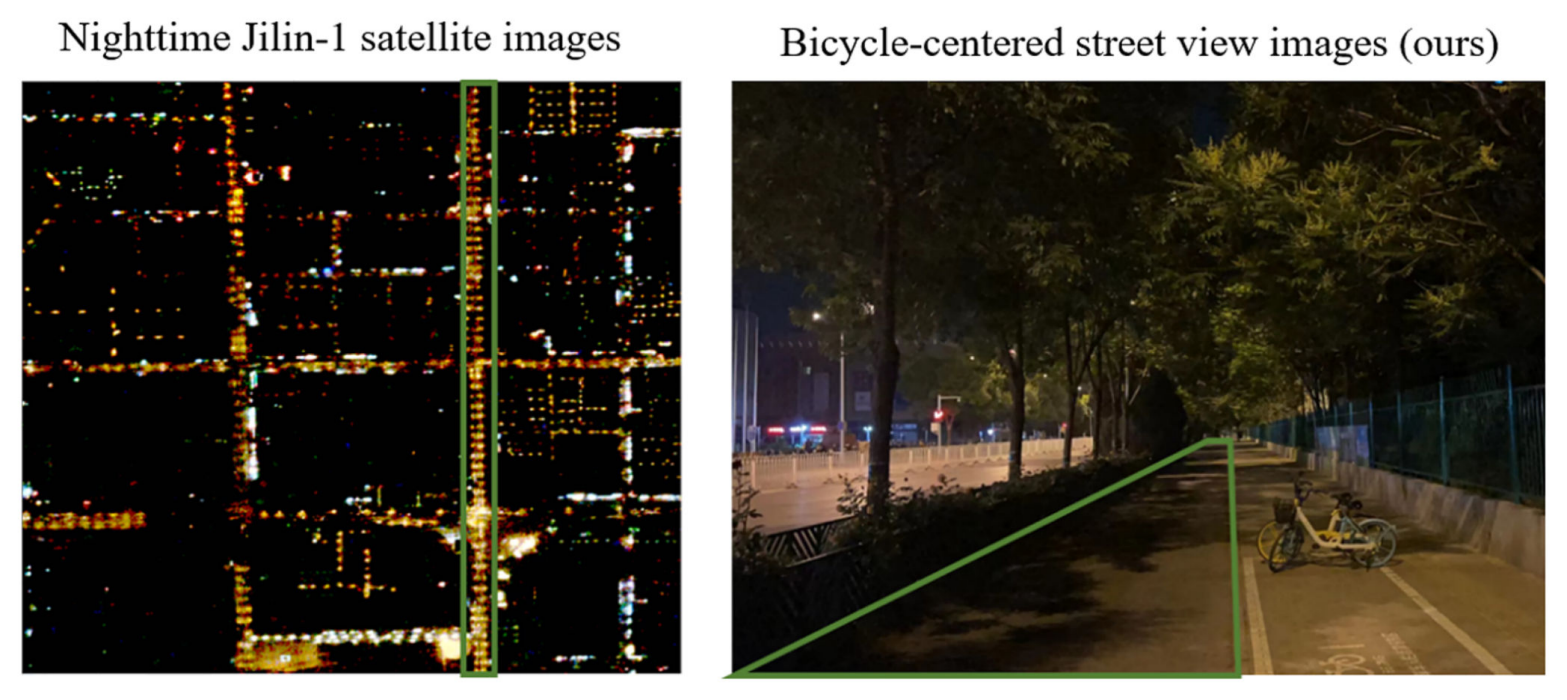
Comparison between nighttime remote sensing images and nighttime bicycle-centered street view images *Image*. Source: Jilin-1 satellite image (left) and mobile sensing image (right)

Past investigations into nighttime lighting assessments have traditionally relied upon methodologies such as evaluating light pole density (Jiang et al., 2018) or analyzing nighttime remote sensing imagery (Levin et al., 2020). Notably, even with the Jilin-1 data, a popular choice among researchers due to its exceptional resolution for identifying nighttime lights in remote sensing data, it remains challenging to capture the lighting conditions within bicycle lanes. This limitation arises because assessments from the sky perspective, characteristic of remote sensing data, fail to reflect the real-world scenario wherein bicycle lanes often remain inadequately illuminated due to tree shading (see Fig. 6).

Furthermore, even within regions boasting a higher concentration of light poles, non-motorized lanes continue to fall short of the illumination standards. This shortfall can be attributed to the obstruction of light by tree trunks, underscoring the significance of factors such as pole height and emphasizing the critical nature of our unique data acquisition perspective.

### 4.3 Policy implications

The establishment of urban slow transportation infrastructure, coupled with its safety enhancement, represents a significant imperative in China. In recent years, there has been a rapid proliferation in the number of electric bicycles, concomitant with a marked escalation in traffic accidents involving such vehicles. The development of infrastructure for slow transportation faces considerable challenges, particularly in nations where electric bicycles coexist with traditional bicycles. A key distinction between electric bicycles and their conventional counterparts lies in their greater dimensions and accelerated speeds, necessitating elevated standards for the cycling environment, including road surface quality and the unlawful use of bicycle lanes. Traditional bicycles exhibit extended reaction times and exert less pronounced effects on road safety. In contrast, electric bicycles engender heightened risks owing to their swifter velocities. Moreover, their larger dimensions elevate the likelihood of encroaching onto motorized lanes, particularly when confronted with narrow or unsegregated bicycle lanes, thereby amplifying the prospect of collisions with motor vehicles.

Nonetheless, the present-day design of bicycle infrastructure predominantly caters to traditional bicycles, neglecting the advantageous road configurations required for electric bicycles. This oversight engenders substantial safety risks and impediments to the advancement of slow transportation. Existing guidelines for bicycle lane design in China, exemplified by documents like the “Urban Road Engineering Design Specification CJJ37-2012” and the “Urban Walking and Bicycle Traffic System Planning Standard GB/T51439-2021”, predominantly furnish guidance pertaining to conventional bicycles. Essential factors including the type and dimensions of bicycle lanes, bicycle lane width, road conditions within bicycle lanes, and other pertinent man-made impediments such as parking and vending, are common precursors to accidents involving electric bicycles. Consequently, the planning and environmental design of bicycle lanes must incorporate the distinctive attributes of electric bicycles into the design and construction processes. The evaluation results can be used to optimize the design of bicycle lanes, improve cycling safety, and enable a shift from motorized transportation to active transportation.

The primary determinants influencing cycling safety include lane type, road conditions, roadside parking, and insufficient nighttime lighting. The most prevalent lane type is painted bike lanes, constituting 53.2 %, while roads lacking any separation facilities account for 4.7 %. To optimize cycling safety and safeguard cyclists’ rights, strategic measures involve judiciously increasing non-isolated facilities given the current road width conditions. Regarding road conditions, the research identified issues such as water accumulation, potholes, and cracks on bike lanes (Table 5). To address these challenges, focused efforts should be directed towards improving road smoothness to ensure a seamless cycling experience. Roadside parking, a significant concern, exhibits an average of 21 parked vehicles per kilometer in the study area, encroaching on bike lanes. Regulatory measures are imperative to address this issue, emphasizing the need to enforce penalties and enhance supervision to deter behaviors that compromise safety, such as motor vehicles parking along bike lanes. In terms of inadequate night-time lighting, suggested solutions include lowering streetlight height to mitigate tree obstruction, replacing outdated fixtures, and installing new lighting facilities. These proactive steps aim to alleviate the problem of insufficient nighttime illumination, enhancing overall safety for cyclists during darker hours.

## 5 Conclusion

In response to the urgent need for safety assessments of bicycle infrastructure in the face of the surging popularity of e-bike and the lack of spatial data, we propose a bicycle-based mobile sensing approach. This method enables the cost-effective, efficient, and large-scale acquisition of day and night streetscape data for a 59.5-kilometer bicycle lane in a rural township in Beijing. We have developed a computer vision-based model for assessing bicycle-related risk factors, and our main findings include the following:

(1) This study addresses a multi-user perspective within bicycle lanes, encompassing both traditional and electric bicycles. It systematically analyzes crucial risk factors associated with bicycle infrastructure and selects 10 factors for empirical research in China.
(2) The dataset obtained from the mobile sensing approach out-performed other data sources, including commercial street view images, nighttime remote sensing data, and OpenStreetMap data, in terms of perspective, spatial coverage, and update time.
(3) The study area is very typical and represents the unsatisfactory bicycle lane situation in Beijing, with half of the lanes having no bicycle lanes or shared bicycle lanes, which are considered unsafe. On-street parking and poor road conditions are quite common in Beijing. Lighting is also unsatisfactory, as most roads are below safety standards. The study has practical implications for bicycle infrastructure improvements.
(4) The study also identified the impact of China’s e-bike boom on bicycle infrastructure as well as bicycle travel safety, and discussed the implications for current bicycle safety policies and road designs.

However, our current study is constrained by the use of camera-only data collection. We acknowledged that future studies should consider incorporating additional sensors and measurement tools to capture a wider range of non-vision features, including more accurate measurements of road friction, road gradients, and other relevant factors. This will set the stage for more comprehensive safety assessments in future research.

We also acknowledge the significance of utilizing more electric vehicles for data collection. Currently, we are limited by our resources, with only two researchers actively participating in this research, which makes it challenging to increase our data collection efficiency. Given that two of the indicators, on-street parking and large vehicles, may vary with traffic flow, we will consider future conditions assuming we have more personnel to cover the entire area in a shorter time.

This study represents an initial phase, requiring formal data collection, model training, and preparation for a larger-scale initiative. Recognizing the importance of employing more electric bicycles, we must balance this with our current staffing situation and available resources. Before embarking on extensive data collection, we need to carefully assess the necessity, considering the significant human and financial resources involved. Our strategy for model training involves utilizing the initially collected data for comprehensive training. Subsequently, we aim to validate and refine the model by using data collected on a larger scale. Drawing from the experience gained during the initial data collection, we aim to determine the optimal number of data collectors required, the time frame for collection, and the overall logistics for extending data collection across the entire Beijing metropolitan area.

## Figures and Tables

**Table 1:** Methods for assessing bicycle lane safety in the literature.

Evaluation Technique	Data	Data Collection Complexity	Data Processing Efficiency	Geographical Scope	Sources
Accident Data Analysis	Incident logs, law enforcement records	High	High	Municipal/National	Siman-Tov et al. (2018)
Conflict Analysis	Field observations, video analytics	High	Moderate	Segment/Intersection	Schleinitz and Petzoldt (2019)
Traffic Simulation	Computational modeling, traffic datasets	High	Moderate	Segment/Intersection	Nazemi et al. (2021)
Cyclist Telemetry Analysis	GPS tracking, sensor telemetry	High	Moderate	Urban/Regional	Saad et al. (2019)
Cyclist Perception Assessment	Surveys, structured interviews	Moderate	Moderate	Urban/Regional	Calvey et al. (2015)
Video-based Analysis	Video captures, software-driven analysis	Moderate	High	Segment/Intersection	Gitelman et al. (2020),Gitelman et al. (2018)
Community Engagement	Stakeholder feedback, community forums	Low	Low	Urban/Regional	Sandt et al. (2015)
Road Safety Audit	Field assessments, design schematics	Low	Low	Segment/Intersection	Nabors et al. (2012)
Street view-based Analysis	Google street views	Low	High	Municipal/National	Ito and Biljecki (2021)

**Table 2:** Bicycle safety risk factors.

Reference samples of bicycle safety risk factors				
**1. Bicycle lane type**				
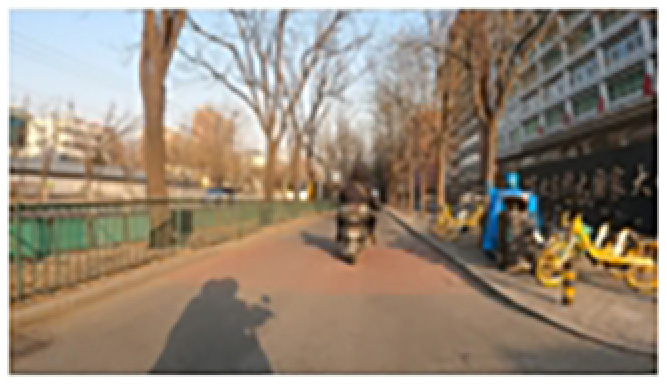 Separate bicycle lane I - Green belts	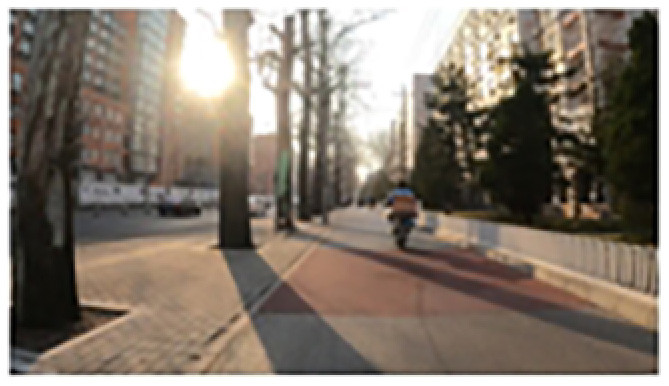 Separate bicycle lane II - Sidewalk	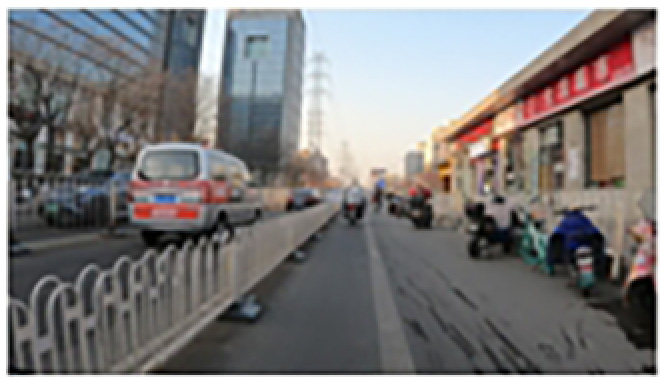 Protected bicycle lane (Guardrail)	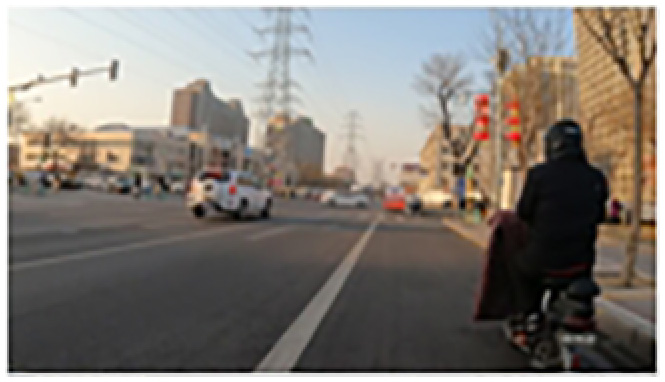 Painted bicycle lane (Lane lines)	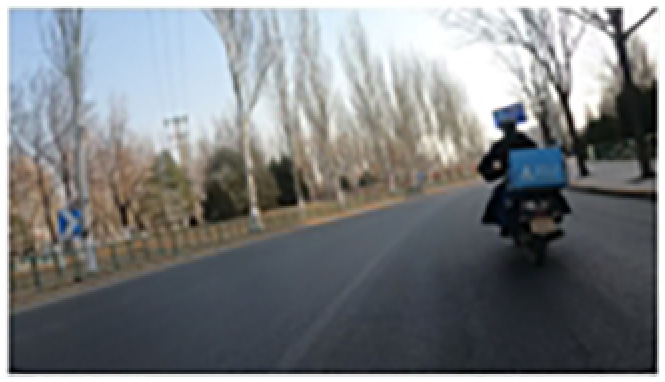 No bicycle lane
**2. Infrastructure**				
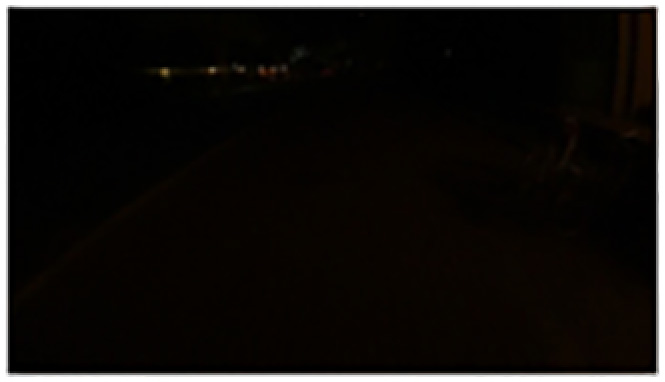 Insufficient lighting	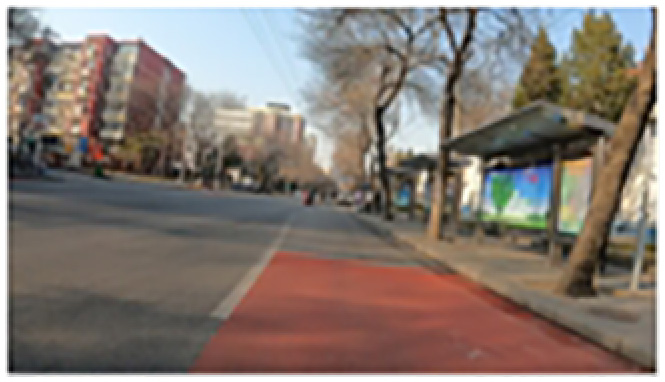 Bus stop presence	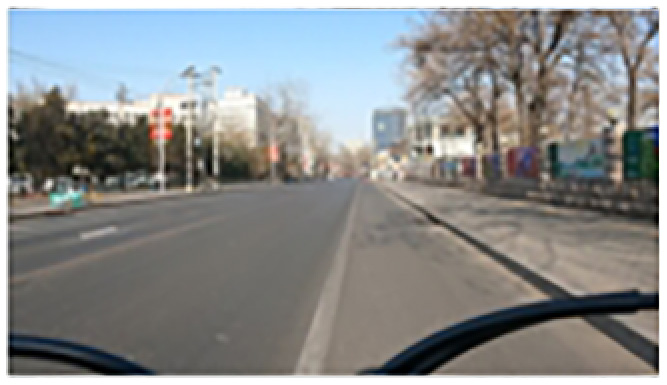 Narrow bike lane	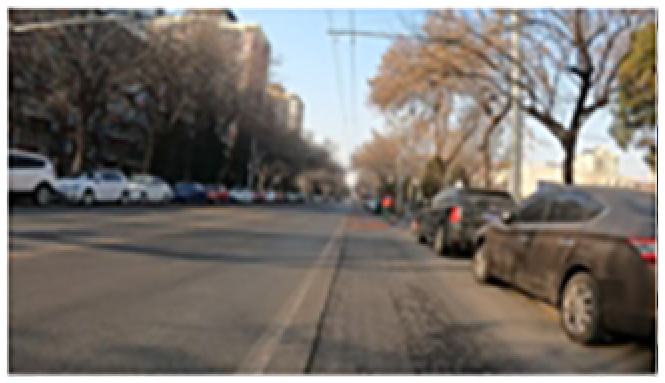 On-street parking	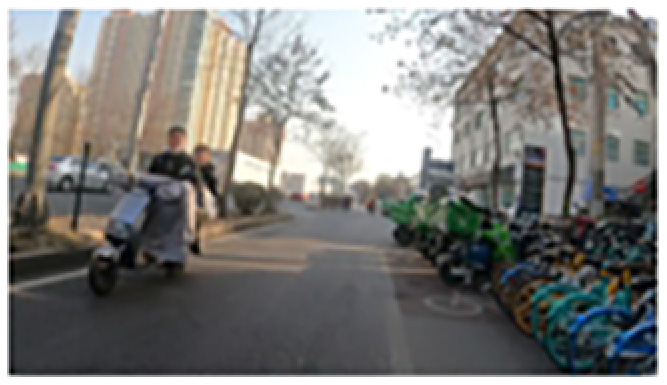 Object blocking bike lane
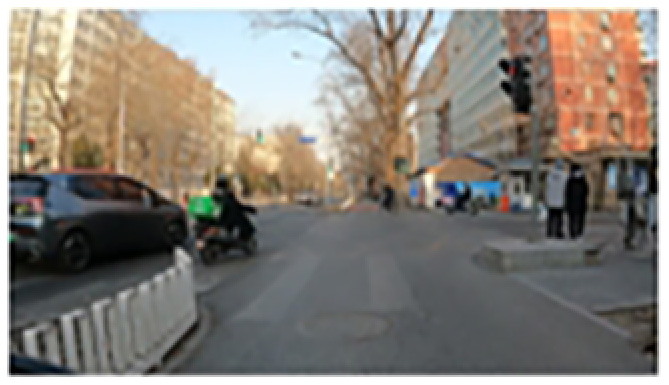 Entrances presence	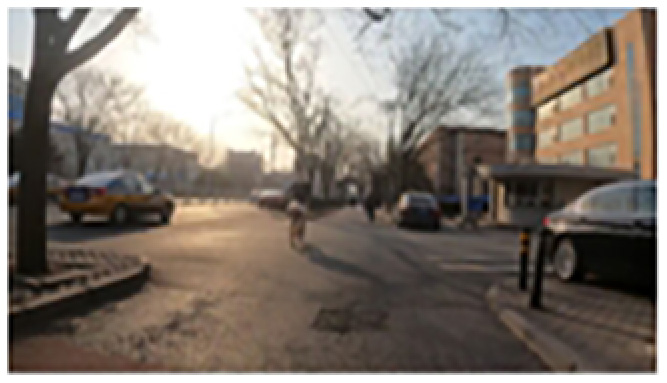 Adverse pavement condition	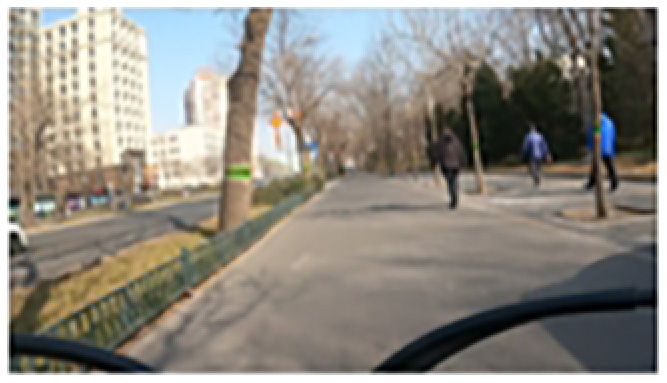 Same height between sidewalk and bike lane	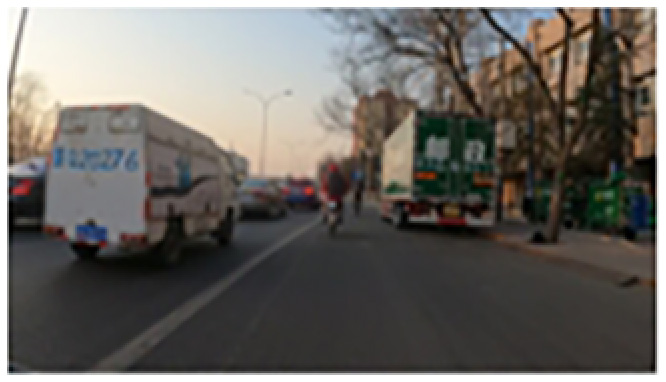 Large vehicle presence	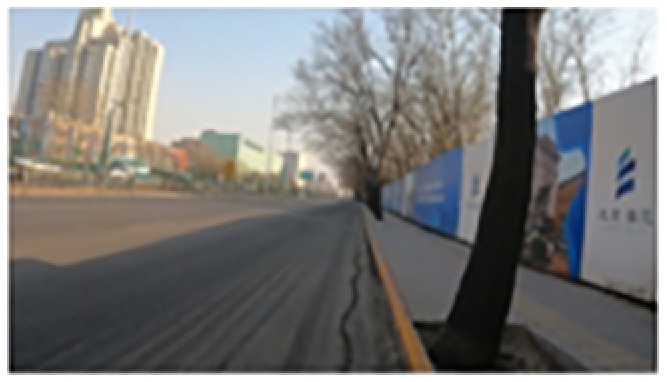 Low road friction
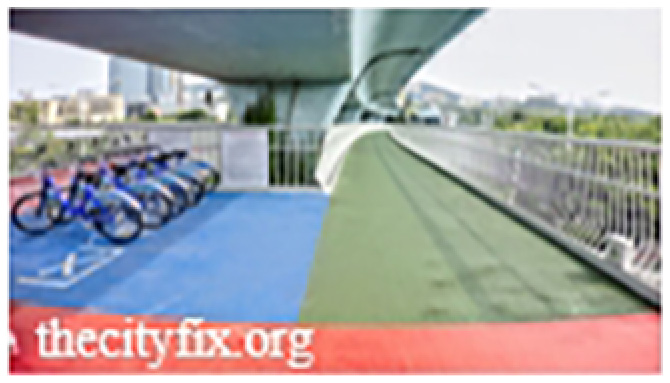 High road gradient	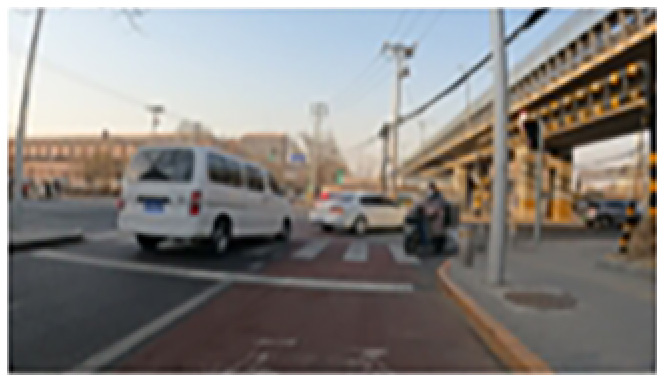 High traffic noise	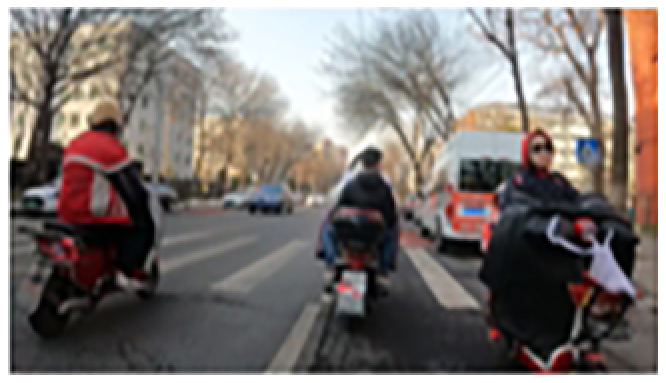 High bicycle traffic demand		
**3. Intersection**				
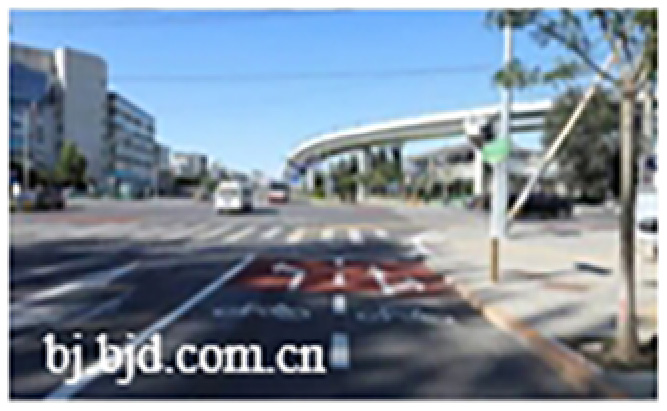 Left-turn Bike Lanes	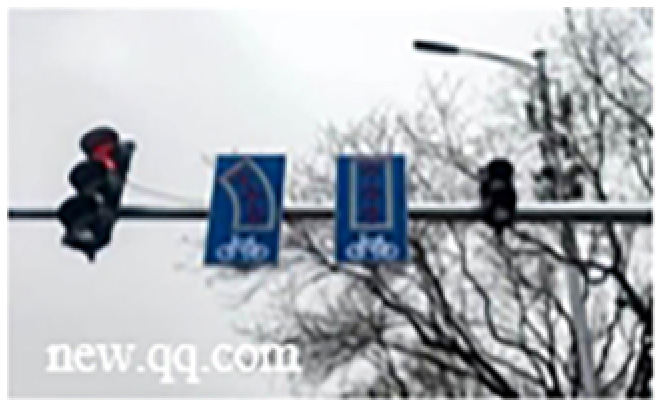 Bicycle-specific traffic signals	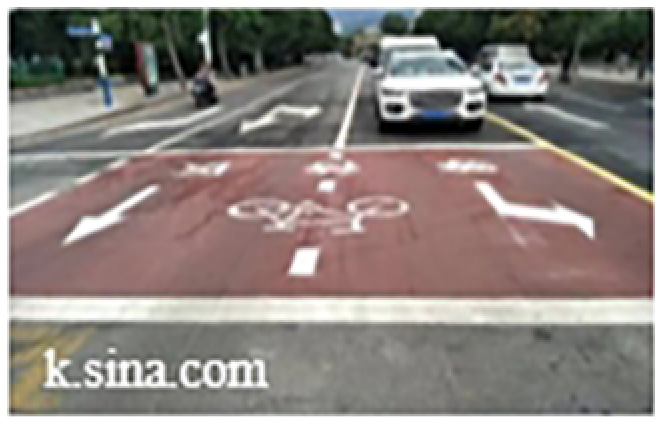 Bicycle waiting areas		

*Image source: Mobile sensing images, except for tags*.

**Table 3:** Measurement of risk factors at street level.

Risk factors	Definition	Method	Calculation
**Bicycle lane type**	Separated bicycle lane	Object detection	Convert lane types to risk levels 0–3
	Protected bicycle lane	Object detection	
	Painted bicycle lane	Object detection	
	No bicycle lane	Object detection	
**Insufficient lighting**	Average luminance below 0.5 cd/m^2^	Luminance calculation	If present, the value is equal to 1; if not, it is 0.
**Bus stop presence**	Bus stop presence on the bicycle lane	Object detection	Quantity per kilometer
**Narrow bicycle lane**	Average bicycle lane width below 1.5 *m*	Lane detection + camera calibration	If present, the value is equal to 1; if not, it is 0.
**On-street parking**	On-street parking	Object detection	Quantity per kilometer
**Object blocking bicycle lane**	Bicycle and construction blocking bicycle lane	Object detection	Quantity per kilometer
**Entrance presence**	Parking lot and building entrances	Object detection	Quantity per kilometer
**Adverse road condition**	Street ponding, potholes, cracks	Object detection	Quantity per kilometer
**Same height between sidewalk and bicycle lane**	Same height between sidewalk and bicycle lane	Object detection	Quantity per kilometer
**Large vehicle presence**	The presence of large vehicles in the bicycle lane or adjacent roads	Object detection	Quantity per kilometer
**High road gradient**	Road gradient reflects the ratio of vertical change to horizontal distance.	GPS based estimation	Percentage
**High traffic demand**	The quantity of bike users	Object detection	Quantity per kilometer
**High traffic noise**	Average traffic noise levels along the road	Random forest model	dB

**Table 4:** Statistics summary of risk factors compared with state-of-art methods.

			Mobile sensing images + machine learning (Our approach)					Baidu street views + machine learning				
	Risk factors		Min	Max	Mean	SD		Min	Max	Mean	SD	
	Bicycle lane type (0–3)		0.000	3.000	1.379	0.822		0	3. 000	1.653	2.119	
	Insufficient lighting (cd/m^2^)		0.000	1.489	0.463	0.485		–	–	–	–	
	Bus stop presence (quantity/km)		0.000	7.827	1.901	3.622		0	9.7	0.132	0.543	
	Bicycle lane width (m)		1.469	10.705	4.419	3.450		0	7.043	1.962	1.239	
	On-street parking (quantity/km)		0.000	87.125	20.681	33.574		0	58.552	23.831	56.965		
	Object blocking bicycle lane (quantity/km)		0.000	20.917	4.904	10.183		0	10.924	4.275	8.652	
	Entrance presence (quantity/km)		0.000	4.755	1.269	1.789		0	30.735	1.458	4.690	
	Adverse road condition (quantity/km)		0.000	50.615	14.478	24.538		0	35.524	0.426	1.731	
	Same height between sidewalk and bicycle lane (quantity/km)		0.000	8.813	2.436	1.393		0	17.132	0.247	1.295	
	Large vehicle presence (quantity/km)		0.000	3.374	1.596	0.496		0	2.853	0.334	1.526	
	High road gradient		0.000	0.085	0.027	0.025		–	–	–	–	
	High traffic demand		0.000	120.000	32.120	20.103		0.000	82.000	32.145	13.856	
	High traffic noise		31.3	98.5	68.7	5.4		–	–	–	–	

**Table 5:** Clustering results of identified risk factors.

Risk factor		Image samples	Categories
**Daytime risk factor**			
**Bicycle lane type**	Separated bicycle lane	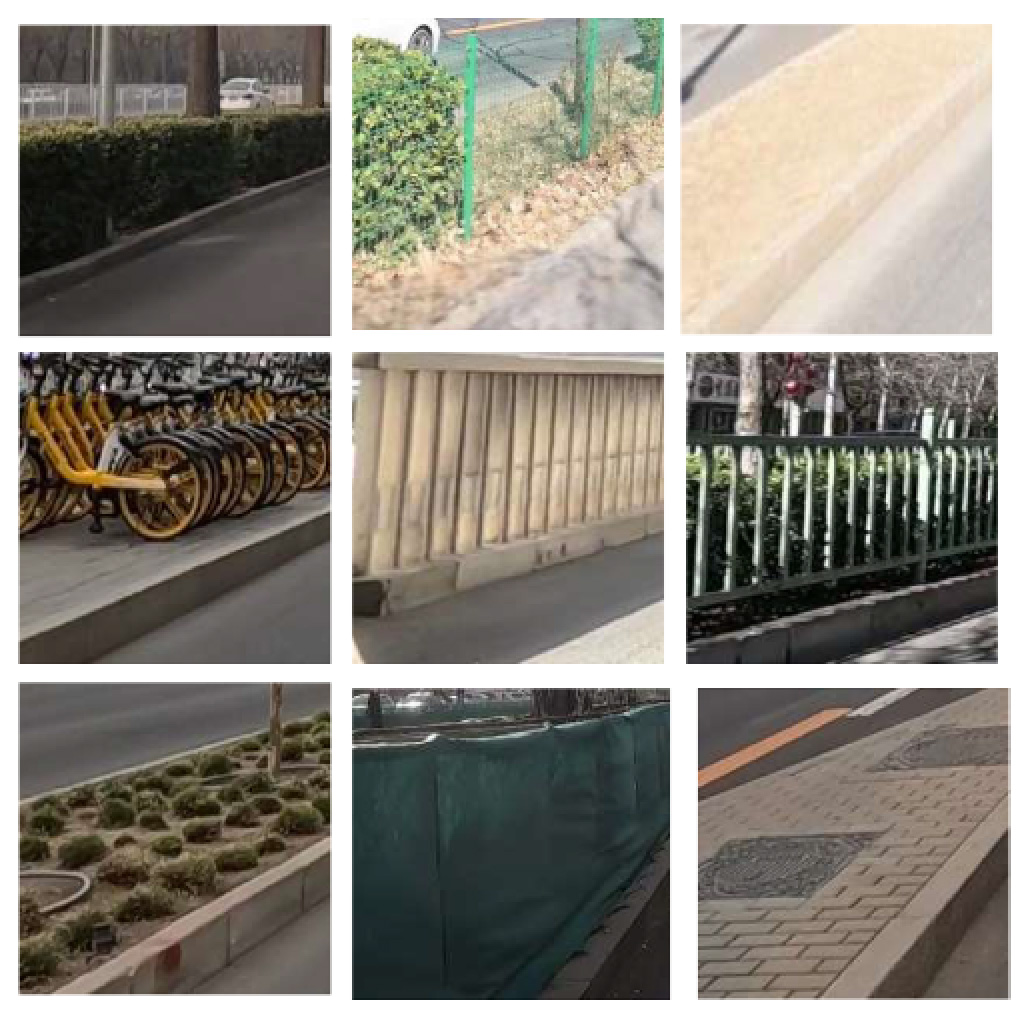	Including shrubbery green belts, green belts with railings, sandy green belts, fences, pedestrian sidewalks with bicycle parking, white railings, green railings, bus waiting areas, and pedestrian crosswalks.
	Protected bicycle lane	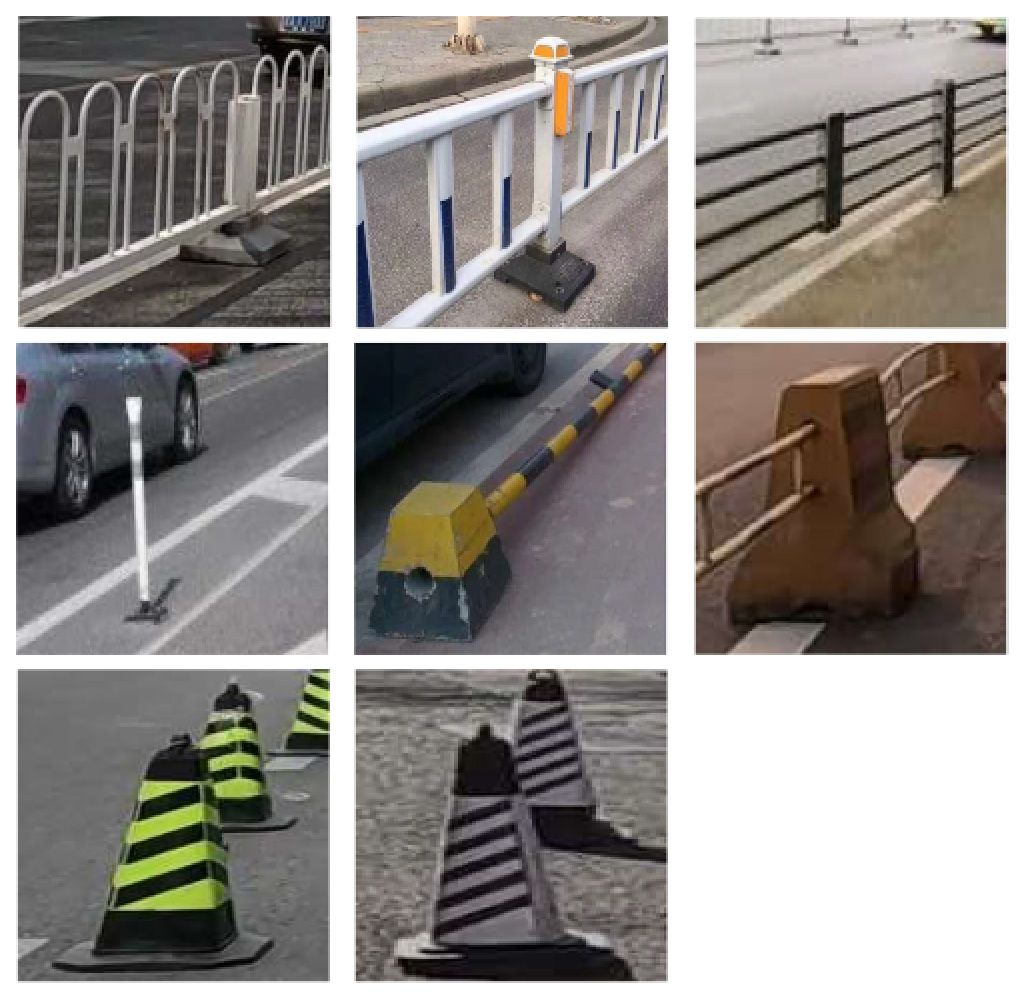	Movable infrastructure such as railings, barriers, and traffic cones.
	Painted bicycle lane	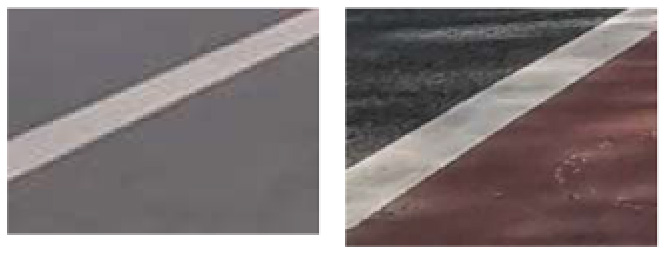	To separate motor vehicle lanes and bicycle lanes with white markings.
**Bus stop presence**		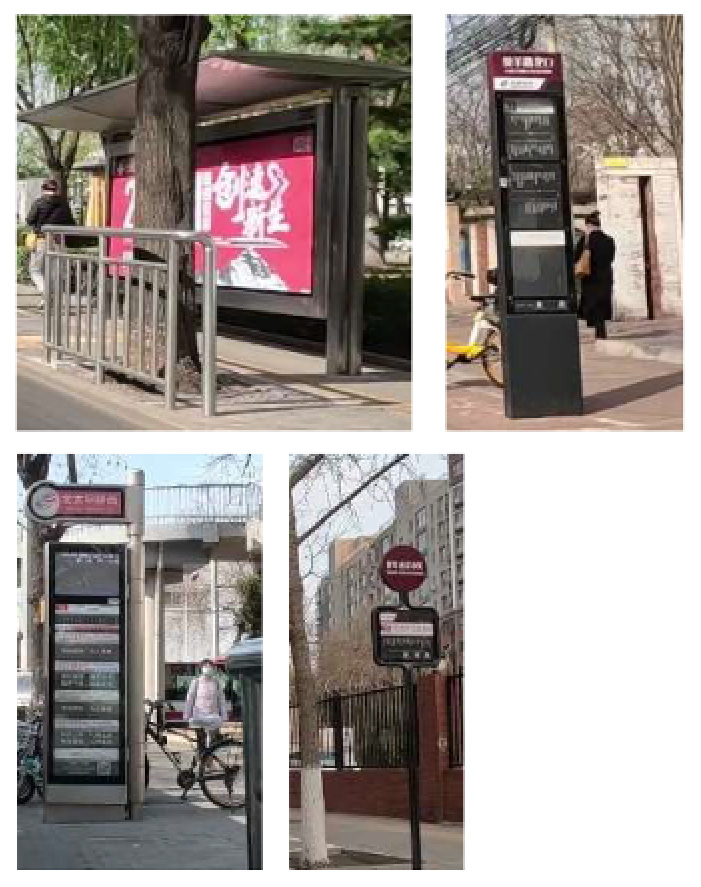	Three types of bus stop signs and bus stop waiting areas.
**On-street parking**		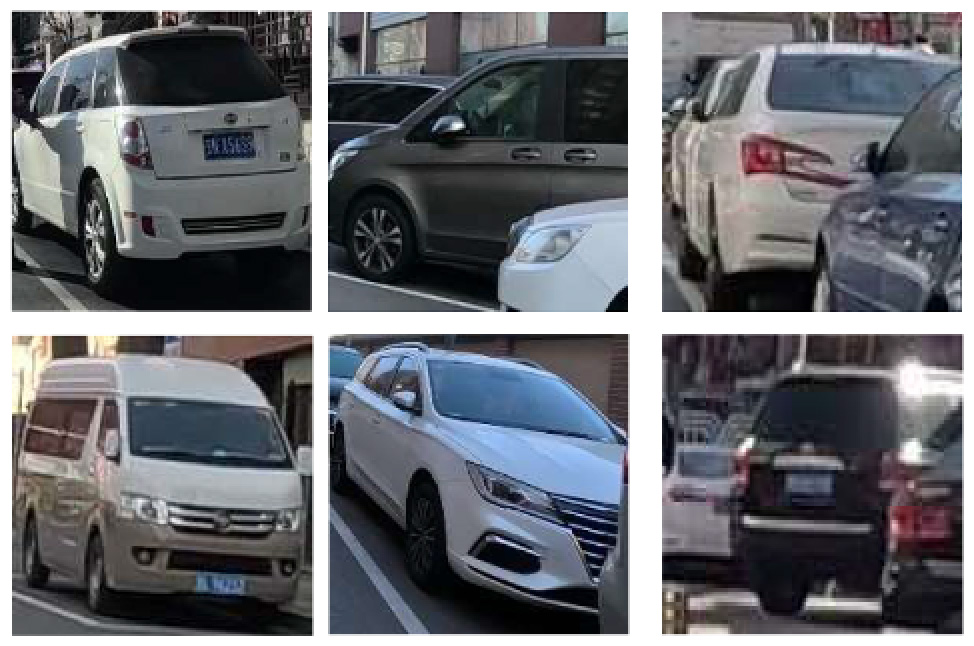	Different types of motor vehicles parked parallel, diagonally, and perpendicularly along the roadside, either facing in the direction of traffic flow or against it.
**Object blocking bicycle lane**		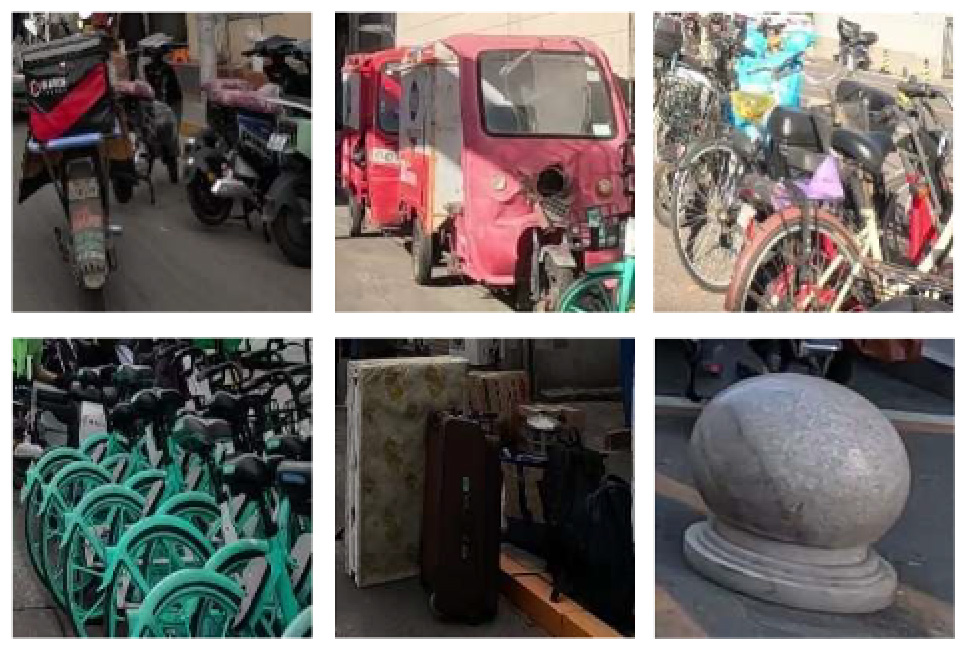	Objects occupying the bicycle lane, such as electric bicycles, delivery tricycles, privately owned bicycles, shared bicycles, personal belongings, fences, discarded items, circular road bollards, and signs.
**Entrance presence**		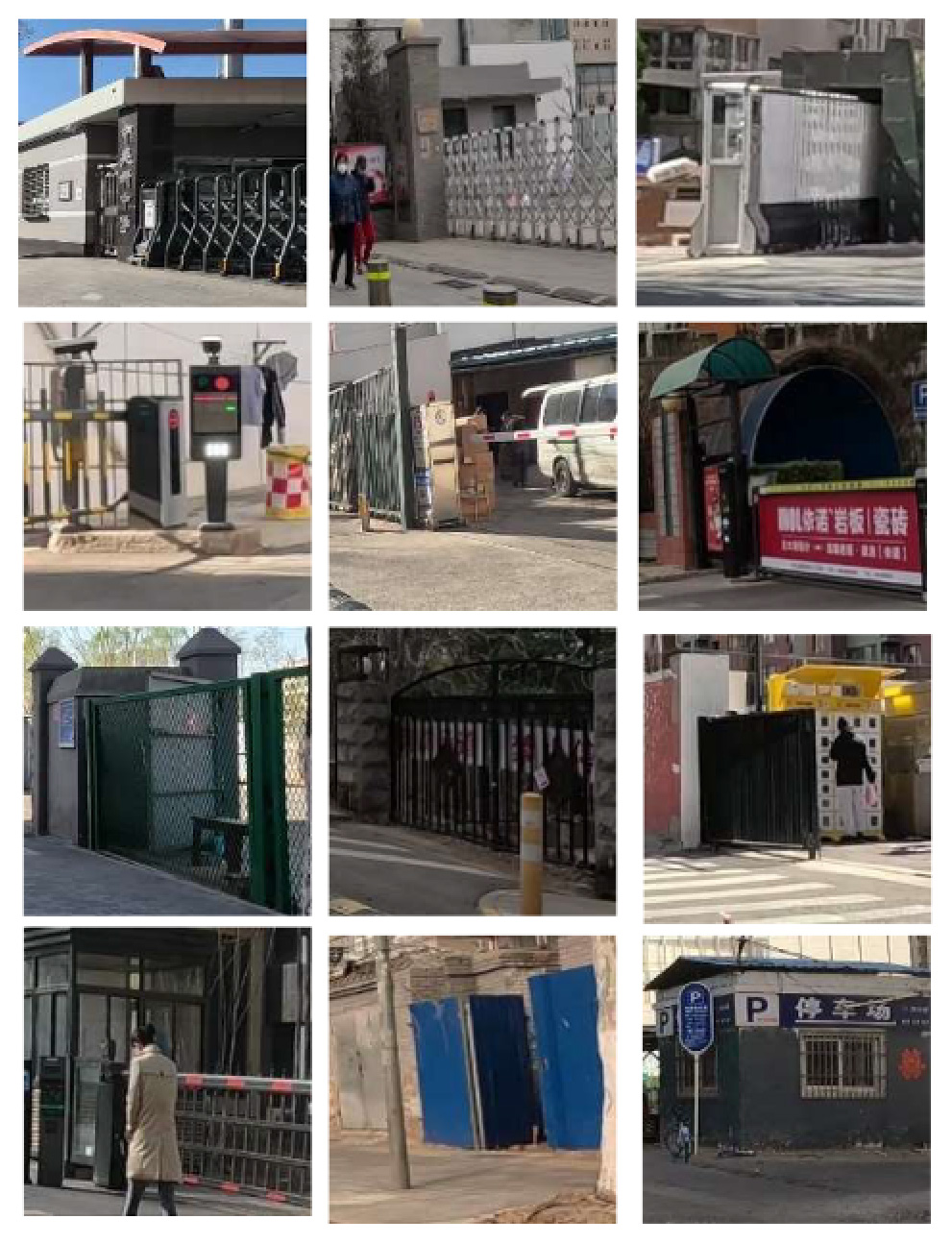	Retractable electric gates, arm vehicle barrier, sliding and swing gates, parking lot signs, iron gates, and other elements found at the exits of residential areas, parking lots, and entrances to office premises where motor vehicles exit.
**Adverse road condition**		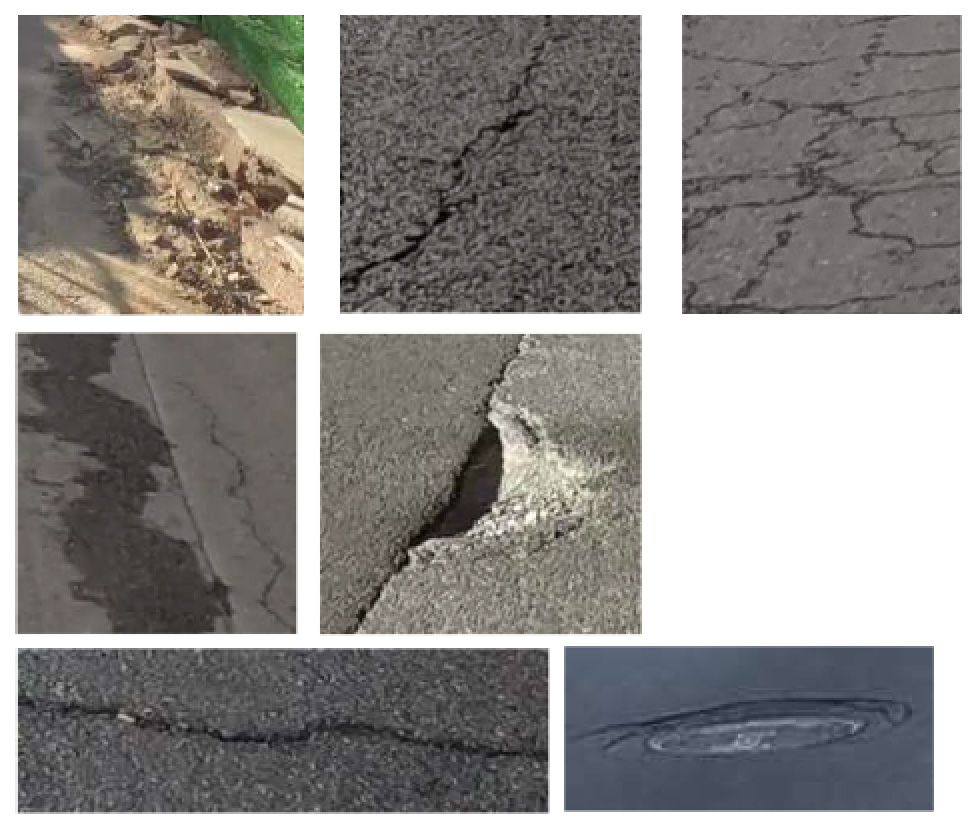	Road surface damage, longitudinal cracks, mesh cracks, transverse cracks, potholes, water accumulation, uneven manhole covers.
**Same height between sidewalk and bicycle lane**		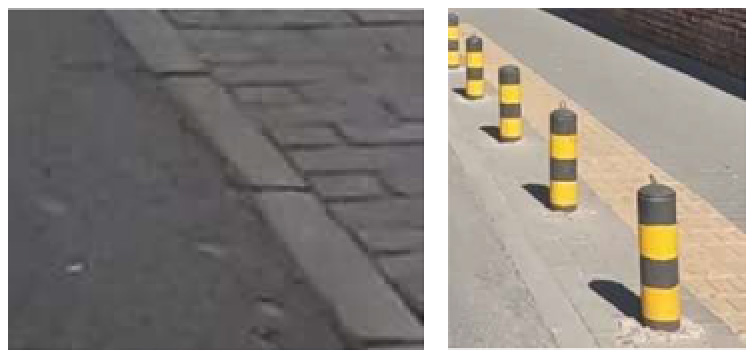	No separation and bollard barriers between sidewalk and bicycle lane.
**Large vehicle presence**		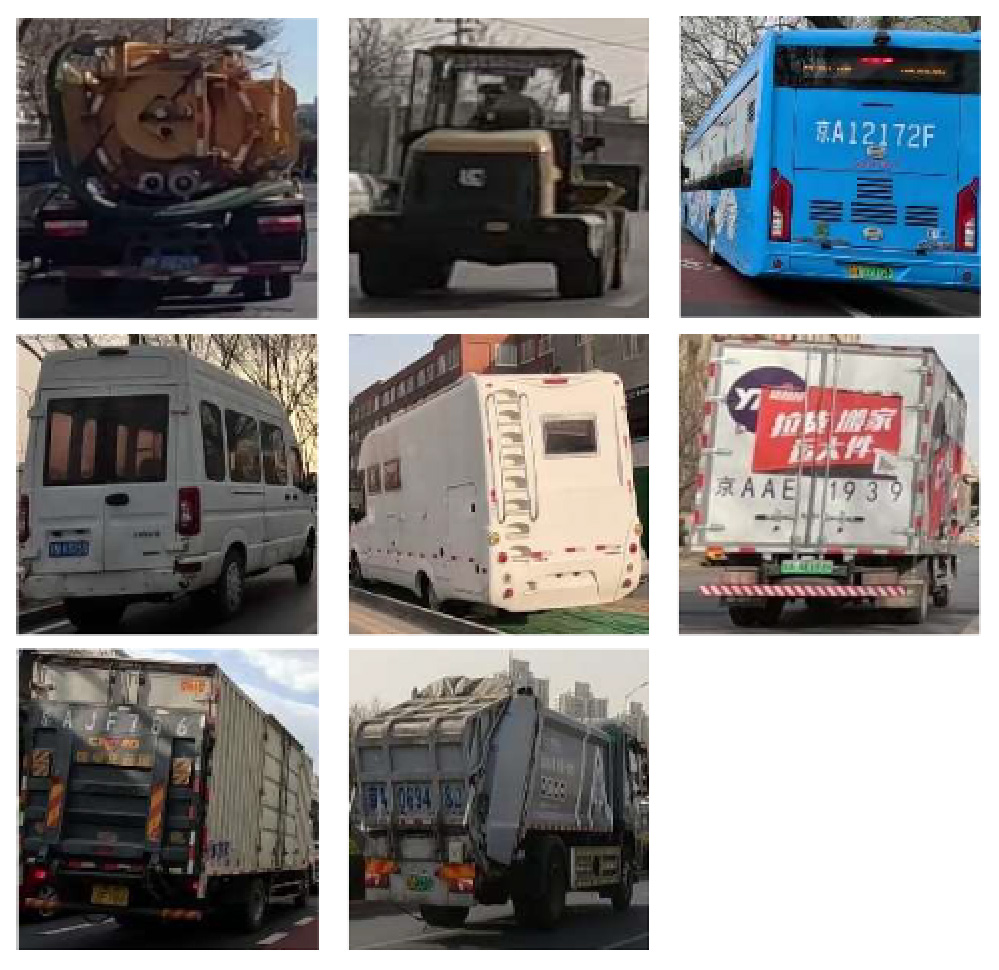	Trucks, municipal vehicles, buses, freight trucks, and recreational vehicles.
**High bicycle traffic demand**		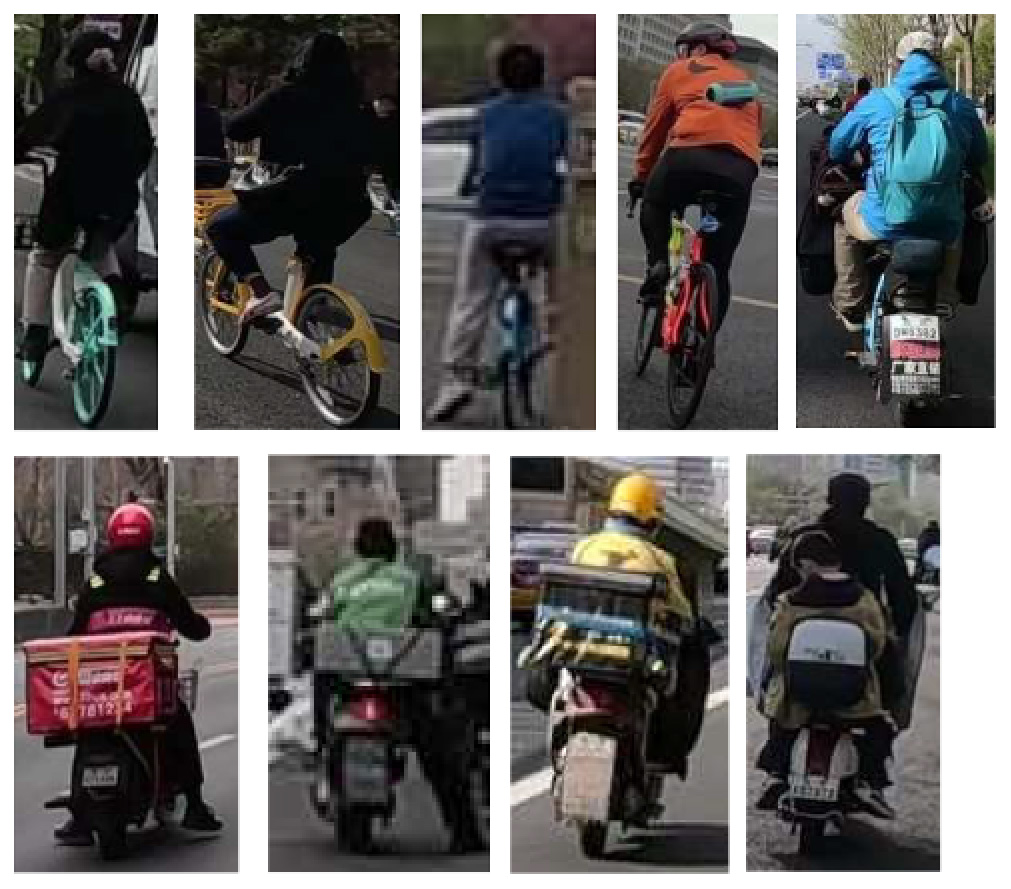	Traditional bicycle riders, shared bike users, personal electric bike riders, and food delivery/courier electric bike riders

**Table 6:** Comparison of Mobile sensing data (ours) and commercial SVI data in the study area.

	Mobile sensing images	Baidu SVI images
No. of sampling points	5770	5770 ( Same as mobile sensing SVs )
Sampling interval	10 m	10 m
Directions	Front	Front
Image size	1920x1080	800x400
Year	2022	Latest ( 2013-2022 )
Perspective	Bike lane	Motorway
Time coverage	Daytime and nighttime	Daytime
